# Prevalence of fermented foods in the Dutch adult diet and validation of a food frequency questionnaire for estimating their intake in the NQplus cohort

**DOI:** 10.1186/s40795-020-00394-z

**Published:** 2020-12-03

**Authors:** Katherine J. Li, Elske M. Brouwer-Brolsma, Kathryn J. Burton, Guy Vergères, Edith J. M. Feskens

**Affiliations:** 1 Division of Human Nutrition and Health, Department of Agrotechnology and Food Science, Wageningen University & Research, P.O. Box 17, 6700 AA Wageningen, Netherlands; 2 Food Microbial Systems Research Division, Agroscope, Federal Office for Agriculture (FOAG), Federal Department of Economic Affairs, Education and Research (EAER), Bern, Switzerland

**Keywords:** Fermented foods; food frequency questionnaire, 24-h recall, Validation, Dietary assessment

## Abstract

**Background:**

Humans have a long history of consuming fermented foods. However, their prevalence in human diets remains largely undetermined, and there is a lack of validated dietary assessment tools assessing the intake of different fermented products. This study aimed to identify fermented foods consumed in The Netherlands and determine the relative validity of a food frequency questionnaire (FFQ) compared to multiple 24-h recalls for estimating their intake.

**Methods:**

The validation population consisted of 809 participants (53.1 ± 11.9 years) from a Dutch observational cohort (NQplus) who completed a FFQ and multiple 24-h recalls. Fermented foods from the FFQ and recalls were identified and aggregated into conventional food groups. Percent difference in mean intakes, quintile cross-classification, Spearman’s correlations, and Bland-Altman analyses were used to evaluate the agreement between the two dietary assessment methods.

**Results:**

Approximately 16–18% of foods consumed by this population were fermented, and a further 9–14% were dishes containing a fermented ingredient. Fermented foods with the highest consumption included coffee (~ 453 g/day;~ 0.5% of daily energy intake), yoghurts (~ 88 g/day;~ 2.2%), beer (~ 84 g/day;~ 1.7%), wholegrain bread (~ 81 g/day;~ 9.4%), wine (~ 65 g/day;~ 2.7%), and cheese (~ 32 g/day;~ 5.0%). Mean percent difference between the FFQ and recalls was small for fermented beverages (coffee), breads (brown, white, wholegrain, rye), and fermented dairy (cheeses) (0.3–2.8%), but large for buttermilk and quark (≥53%). All fermented food groups had > 50% of participants classified into the same or adjacent quintile of intake (58%-buttermilk to 89%-fermented beverages). Strong Spearman’s correlations (crude/energy-adjusted r_s_ ≥ 0.50) were obtained for fermented beverages (coffee, beer, wine), cereals/grains (wholegrain bread), and dairy (yoghurts). For ‘other bread’, quark, and buttermilk, correlations were low (r_s_ < 0.20). Bland-Altman analyses revealed good agreement for fermented beverages (coffee, beer), breads (brown, wholegrain, rye, other), pastries, chocolate, and fermented dairy (cheeses) (mean difference: 0.1–9.3).

**Conclusions:**

Fermented food groups with acceptable or good validity across all measures included commonly consumed foods in The Netherlands: fermented beverages (coffee), wholegrain and rye bread, and fermented dairy (cheeses). However, for less frequently consumed foods, such as quark and buttermilk, the levels of agreement were poor and estimates of intake should be interpreted with caution. This report provides the basis for developing a FFQ specific for fermented foods.

**Supplementary Information:**

The online version contains supplementary material available at 10.1186/s40795-020-00394-z.

## Background

Fermented foods are foods or beverages in which microorganisms have been intentionally added or used to enzymatically transform food components [1]. They comprise a large, pervasive group of foods in the Western diet, including cheese, yoghurt, buttermilk, coffee, beer, wine, bread, sauerkraut, dried sausages, and chocolate. The fermentation process not only improves the shelf-life and organoleptic qualities of a food, but it can also impart novel nutritional qualities through the introduction of live microorganisms and/or bioactive compounds generated via microbial action [2]. Several studies have associated the consumption of fermented foods with positive impacts on cardiometabolic health outcomes, including improvements in body weight, modulations in blood cholesterol, and prevention of type II diabetes [3–7]. However, assessment of the true intake of fermented foods is limited due to the subjective nature of many traditional dietary assessment tools (that are also not specific for assessing fermented food intake), and the lack of validation of these methods for assessing the intake of different fermented food groups.

Accurate dietary assessment is a core tenet of nutritional epidemiology that aids in the appropriate identification of diet-health associations. To date, the food frequency questionnaire (FFQ) is one of the most common dietary assessment instruments used to estimate habitual food and nutrient intake in large populations, for reference periods of 1 month to 1 year [8]. Since the FFQ food list is determined based on the major foods that contribute to the total intake, as identified in food consumption surveys, it is not necessarily designed to assess the total diet [8–10]. Conversely, 24-h recalls aim to assess the whole diet, but only for the previous 24 h prior to assessment [11]. In theory, multiple, non-consecutive 24-h recalls can approximate habitual intake of a food or nutrient, akin to the FFQ, but this process can be labour-intensive. Both methods rely on self-reporting, and are prone to correlated measurement and reporting errors. Nevertheless, determining the level of agreement between intakes assessed by the FFQ versus multiple 24-h recalls may provide a better approximation of ‘true’ dietary intake. This could help with the interpretation of the results in future studies, and avoid misidentified associations between dietary components and health.

The validity of FFQs in estimating intakes of various nutrients, foods, and food groups has been documented in multiple studies [12–16]. However, to our knowledge, no groups have endeavoured to assess the validity of FFQs for estimating the intake of fermented foods. In this study, we aimed to first identify fermented foods in the diet, and subsequently assess the relative validity of a FFQ compared to multiple 24-h recalls in estimating the intake of fermented foods in a subsample of participants from a Dutch observational cohort study (NQplus). Given that the goal of nutritional epidemiological studies is to identify associations between food intake and the development of chronic diseases, the accurate assessment of dietary intake and classification of individuals into their relative levels of dietary intake is critical in order to promote accurate estimation of risk and prevent false associations.

## Methods

### Participants

The Nutrition Questionnaires plus (NQplus) study is a prospective cohort study that was primarily conducted in Caucasian Dutch adults (20 to 70 years), living in or around Wageningen, The Netherlands. It was initiated as an ‘add-on’ study to the National Dietary Assessment Reference Database (NDARD) project, to gather extensive data on participant demographics, lifestyle, medical history, and cardiometabolic health outcomes. A complete description of NQplus and the NDARD project can be found elsewhere [17, 18]. Briefly, 2048 men and women were recruited and included in the study between June 2011 and February 2013. Baseline measurements included an assessment of habitual dietary intake by FFQ (*n =* 1468) and/or 24-h recall (*n =* 1117). Additional data on anthropometrics, body composition, blood pressure, pulse wave velocity, advanced glycation endproduct (AGE) accumulation, and cognitive performance, were also collected. Background demographics, health, and lifestyle data were collected via validated questionnaires administered online using the open-source survey tool Limesurvey (Lime-Survey Project Team/Carsten Schmitz, Hamburg, Germany). Fasting blood samples and 24-h urine samples were also collected. All measurements were repeated at 1 and 2 years of follow-up and performed according to a standardised protocol by trained research personnel. The study was approved by the ethical committee of Wageningen University and Research and performed in agreement with the Declaration of Helsinki. Written informed consent were obtained from all participants prior to the start of the study.

### Population for the validation study

The validation analyses were conducted with a subset of participants who had completed both a FFQ as well as 2 or more 24-h phone-based recalls. From the original dataset (*n* = 2048), participants who did not have any dietary assessment data were excluded (*n* = 17), as were those who completed fewer than two phone-based recalls (*n* = 1081). A further ten participants with implausible energy intakes were excluded from analyses (i.e., men with energy intakes < 800 or > 4200 kcal/day, and women with energy intakes < 500 and > 3500 kcal/day) [19–22]. Merging the FFQ and 24-h recall data subsets resulted in a sample of *n* = 809 with complete data; these participants represented the validation subcohort for further analyses.

### Food frequency questionnaire (FFQ)

A full description of the dietary assessment methods have been detailed previously in the study design papers for the NQplus study and NDARD project [17, 18]. The goal of NDARD was to advance the development and validation of new FFQs, while NQplus promotes research activities between dietary determinants and cardiometabolic health in Dutch adults. Habitual dietary intake was assessed using a 216-item FFQ. The food items for the FFQ were selected to cover ≥96% of the absolute level of food intake and ≥ 95% of the between-person variability of each nutrient under study as assessed in the 1998 Dutch National Food Consumption Survey (DNFCS), and supplemented with commonly consumed commercial food products from the 2011 DNFCS [17]. The FFQ was self-administered and completed online using the open-source survey tool Limesurvey, with 10 frequency categories: never, 1 day per 4 weeks, 2–3 days per 4 weeks, 1 day per week, 2 days per week, 3 days per week, 4 days per week, 5 days per week, 6 days per week, and 7 days per week. Portion sizes were estimated using typical portion sizes and commonly used household measures. Subsequently, total food intakes (in g/day) were calculated by multiplying consumption frequency (times/day) by portion size (in grams) as defined in the Dutch food composition tables (2011) [23]. It should be noted that although the reference period of the FFQ validity is 1 month, it was assumed that food consumption patterns are stable in this adult population. Previous validation studies for this FFQ have revealed good correlation coefficients for energy (Pearson’s r = 0.65 compared to 24-h recall) [24], total fats (Pearson’s r = 0.78 compared to dietary history) [25], as well as several micronutrients (e.g., vitamin B1 and B2, Pearson’s r = 0.58) and food groups (e.g., bread, Pearson’s r = 0.69) compared to the 24-h recall [15]. In addition, a recent validation study evaluating a Glycaemic Index FFQ (GI-FFQ) against the general-FFQ and 24-h recalls for the NQplus cohort revealed moderate to good relative validity for carbohydrates, carbohydrate-rich foods, and glycaemic index/glycaemic load [26].

### 24-h recalls

For the current analyses, we used 24-h recall data collected by telephone. The telephone-based 24-h recalls were carried out by trained dietitians and performed according to a standardised protocol [17]. Portion sizes were assessed using household measures, weight/volume, and standard reference portions. Recall data were subsequently transcribed as food codes of the 2011 Dutch food composition table [23]. Regular meetings with all dietitians and quality checks ensured the quality of the telephone recalls and encoding of the data. Further information on dietary supplement intake and whether a dietary regime was followed during the month preceding the recall assessment (prescribed or at own initiative) were also recorded. The phone-based 24-h recalls were taken at the beginning of the study period, and at 6, 12, 24, and 36 months follow-up, with some participants completing less or more recalls than the indicated follow-up periods. Participants included in the validation study (*n* = 809) completed between two and eight phone-based 24-h recalls assessing the intake of 2102 food items. The number of participants who completed 2, 3, 4, 5, 6, 7 and 8 recalls were respectively, *n* = 48, 358, 53, 229, 96, 21, and 4.

### Identification and classification of fermented foods

Fermented foods from the FFQ and 24-h recall food lists were identified and classified. As a first step, foods that were not consumed by any participants were removed from the analyses. This left 216 foods in the FFQ food list, and 1593 foods in the 24-h recall food list. To take into consideration the breadth of fermented foods that exist in the marketplace and in the diet, we first stratified fermented foods in the FFQ and 24-h recall food lists into broad food groups, namely dairy, meat and fish, fruits and vegetables, soya, cereals and grains, beverages, and ‘other fermented products’. These food groups were defined a priori and were loosely based on the food-based dietary guidelines in The Netherlands, Switzerland, and United States [27–29]. Fermented foods within each food group were then aggregated into subgroups. To ensure that the foods were truly fermented, a series of exclusion criteria were applied. For foods that were traditionally fermented but are typically no longer fermented due to modern food processing (e.g., pickled vegetables), ingredient lists of common grocery store items were consulted, and these foods were included/excluded accordingly. Foods that contained a fermented ingredient (e.g., composite dishes, such as pizza with cheese, chocolate-based confectionaries), processed variations of fermented foods (e.g., chocolate spreads, cheese spreads), and foods that were not fully fermented (e.g., green or black teas that are usually oxidised rather than post-fermented) were classified separately, as ‘composite dishes that contain a fermented ingredient’ or ‘possibly fermented’.

For the validation aspects of this study, we selected fermented foods and food groups that were assessed by both the FFQ and 24-h recall methods, to enable a direct comparison between the two methods. These fermented food groups (and subgroups) included: fermented beverages (coffee, beer, and wine), fermented cereals/grains (brown bread, white bread, wholegrain bread, rye bread, or ‘other bread’), fermented dairy (cheese, yoghurt, buttermilk, quark), and chocolate. Additionally, we assessed the intakes of non-fermented dairy (milk, ice cream, butter, cream) and non-fermented soya products. Intakes of these products may be closely related to the intakes of the fermented foods and thus were considered as potentially relevant for future analyses wherein associations between fermented food intake and health will be explored.

### Statistical analysis

For the recalls, intakes from the total number of recalls per participant (ranging from 2 to 8) were averaged prior to statistical analysis. We calculated both absolute as well as energy-adjusted intakes for food groups, where energy-adjustment was performed using the commonly used residual method [30]. In order to provide comprehensive insight into the different aspects of validity, and to reveal the limitations of each dietary assessment method, a combination of statistical tests were used to assess relative validity [31]: mean percent difference, quintile cross-classification, correlation coefficient (and attenuation factors), and Bland-Altman. Group-level agreement was first assessed using mean percent difference in energy-adjusted food intake, which was calculated according to the formula:

$$ Difference\ \left(\%\right)=\frac{FFQ- Recall}{Recall}\ast 100 $$

To assess the level of agreement between intakes assessed by the two methods, quintile cross-classification was applied to the mean energy-adjusted intakes for each fermented food group. After defining the quintiles for each food group, the percentage of individuals classified into the same, adjacent, or extreme quintile for each fermented food group was examined. If more than 50% of the participants were correctly classified in the same or adjacent quintile, with less than 5% grossly misclassified in the extreme quintile, this was interpreted as a good outcome [31, 32].

To determine the strength and direction of the associations, non-parametric Spearman’s rank correlation coefficients (r_s_) were calculated; correlations are shown as crude and energy-adjusted. Correlations coefficients of ≥0.50 were classified as good, 0.20 to 0.49 was considered acceptable, and < 0.20 considered as poor [31]. While these cut-offs are commonly used, for the ‘acceptable’ classification, we distinguished between a higher range (0.40 to 0.49) and lower range (0.20 to 0.39), where the higher acceptable range was considered a more rigorous cut-off to take into account the high possibility of correlated errors between the FFQ and recall methods. Attenuation factors were also calculated alongside correlation coefficients, since they are commonly used in epidemiological studies to adjust the association between diet and disease, and help indicate the extent to which diet-disease associations are weakened due to measurement error. Due to the high probability of correlated errors between the FFQ and 24-h recall methods, the attenuation factors are expected to give an incomplete correction of measurement error, and can be inflated [33, 34]. Nevertheless, the use of attenuation factors (based on a 24-h recall method) has been shown to improve the relative risks of diet-disease associations [35], which warranted their inclusion in our analyses. A non-linear mixed model was used to obtain attenuation factors for all food groups. From the model parameters, we calculated the attenuation factors (λ_x_) using the 24-h recall as a reference method according to methods previously described by Trijsburg et al. [36], and specified in the formula:

$$ {\lambda}_X=\frac{\beta_x\ast varT}{{\beta_x}^2\ast varT+\frac{\mathit{\operatorname{var}}\ {\varepsilon}_{Xij}}{k}+{varw}_{xi}} $$

where *β*_*X*_ is the proportional scaling bias of the reference method (*X*), varT is the variance of the true intake, *varε*_*Xij*_ is the variance of the random error of the reference method, and *varw*_*xi*_ indicates the variance of the person-specific bias of the reference method. To obtain the estimates of the attenuation factor for multiple 24-h recalls, the variance of the random error of the method (*varε*_*Xij*_) was divided by the number of measurements (*k*) of the reference method.

Finally, Bland-Altman plots were constructed to examine the group-level agreement between the FFQ and recall (i.e., mean of multiple recalls) by plotting the mean measure [(FFQ + Recall)/2] against the difference in measures (FFQ-Recall) [37]. To visually assess the degree of error, additional analyses were added to the plots, including: a line indicating the mean difference, and upper and lower 95% confidence intervals [mean ± (standard deviation of the mean difference*1.96)]. Additional regression analyses were conducted to detect proportional biases, and evaluate the direction and magnitude of the bias. All analyses were conducted in R, version 3.5.0 [38], with the exception of quintile cross-classification and Bland-Altman analyses, which were conducted using the statistical package IBM SPSS Statistics for Windows, version 22.0 (IBM Corp., Armonk, N.Y., USA), and the attenuation factors, which were calculated using SAS, version 9.3 (SAS Institute Inc. Cary, NC, USA, 2012). The level of statistical significance was set as *p* ≤ 0.05.

## Results

### Participants in the validation study

The characteristics of the participants included in the validation study are shown in Table 1. Participants had a mean age of 53 ± 12 years, and 53% of the population were men. Approximately 48% of participants (39% of men and 58% of women) had a body mass index (BMI) below 25 kg/m^2^, while 52% were overweight or obese (61% of men and 42% of women). The majority of participants had a high educational level (65%), had never smoked (52%), and had not followed a diet in the month preceding enrolment to the study (93%). About a fifth of participants had a disease history of hypertension (22%) and high cholesterol (18%), while only a small percentage (less than 5%) had a history of cancer, diabetes, heart attack, and/or stroke.

**Table 1:** General Characteristics of the Participants Included in the Validation Study

	All (***n*** = 809)	Men (***n*** = 425)	Women (***n*** = 384)
Age, years	53.1 ± 11.9	55.5 ± 11.0	50.4 ± 12.2
BMI, kg/m^2^	25.6 ± 3.8	26.1 ± 3.3	25.0 ± 4.2
BMI category			
< 25 kg/m^2^	387 (48)	165 (39)	222 (58)
≥ 25 kg/m^2^	421 (52)	259 (61)	162 (42)
Waist circumference, cm	90.7 ± 12.0	95.9 ± 10.3	84.8 ± 11.1
Education, n (%)			
Low	54 (7)	31 (7)	23 (6)
Intermediate	223 (28)	109 (26)	114 (30)
High	529 (65)	285 (67)	244 (64)
Smoking status, n (%)			
Never	364 (52)	170 (45)	194 (60)
Former	270 (39)	163 (44)	107 (33)
Current	65 (9)	41 (11)	24 (7)
Disease history, n (%)			
Cancer	44 (5)	17 (4)	27 (7)
Diabetes	21 (3)	15 (4)	6 (2)
Heart attack	16 (2)	12 (3)	4 (1)
Hypertension	179 (22)	101 (24)	78 (20)
High cholesterol	147 (18)	92 (22)	55 (14)
Stroke	9 (1)	8 (2)	1 (0)
Diet during month preceding study, n (%)			
No	749 (93)	402 (94)	347 (91)
Yes, always	28 (3)	8 (2)	20 (5)
Yes, sometimes	31 (4)	15 (4)	16 (4)
Energy, kcal/day			
FFQ	2143.7 ± 504.8	2344.6 ± 509.7	1921.3 ± 394.5
24-h Recall^a^	2129.2 ± 444.2	2315.2 ± 451.2	1923.4 ± 331.5
Protein, g/day			
FFQ	77.4 ± 17.7	83.4 ± 17.7	70.7 ± 15.1
24-h Recall^a^	82.6 ± 18.4	89.8 ± 18.6	74.6 ± 14.5
Fat, g/day			
FFQ	85.4 ± 25.9	93.1 ± 27.1	76.9 ± 21.6
24-h Recall^a^	81.6 ± 21.7	87.7 ± 22.8	74.7 ± 18.1
Carbohydrates, g/day			
FFQ	231.6 ± 61.1	251.7 ± 63.5	209.4 ± 49.6
24-h Recall^a^	230.8 ± 58.0	249.1 ± 62.2	210.5 ± 44.8
Fibre, g/day			
FFQ	25.0 ± 6.8	26.1 ± 7.3	23.8 ± 5.9
24-h Recall^a^	23.4 ± 6.8	24.5 ± 7.1	22.1 ± 6.2

*BMI* Body mass index, *FFQ* Food frequency questionnaire, *SD* Standard deviation

Values are presented as mean ± SD, unless otherwise specified. Missing values: BMI (*n =* 1), waist circumference (*n =* 1), education (*n* = 3), smoking status (*n =* 110), diet during last month (*n =* 1)

^a^Mean of multiple 24-h recalls

### Identification of fermented foods in the diet and comparison of mean intakes

The identification and classification of fermented foods from the FFQ and 24-h recall into food groups and subgroups, is provided in Additional file 1, Table S1. For the FFQ, 39 foods (18%) were classified as fermented, including 5 types of fermented beverages, 12 types of fermented cereals/grains, 3 types of chocolate, 17 types of fermented dairy products, and 2 other fermented products. A further 19 (9%) of foods in the FFQ food list were classified as ‘composite dishes that contain a fermented ingredient’ or ‘possibly fermented’. For the 24-h recall, 247 foods (16%) were classified as fermented, including 20 types of fermented beverages, 95 types of fermented cereals/grains, 20 types of cocoa products, 96 types of fermented dairy, 4 types of fermented fruits/vegetables, 6 types of fermented meat/fish, 4 types of fermented soya, and 2 other fermented products. A further 228 (14%) of foods in the recall food list were classified as ‘composite dishes containing a fermented ingredient’ or ‘possibly fermented’.

Mean energy-adjusted daily intakes and percentage of average daily energy intake for each fermented food group, the number of consumers per food group, as well as the percent and absolute differences in mean intakes, are presented in Table 2. The mean daily energy intake as estimated by the FFQ was 2144 (±505) kcal/day, which was comparable to the energy intake estimated by the 24-h recalls of 2129 (±444) kcal/day (0.68% difference). Fermented food groups with the highest intakes for both the FFQ and 24-h recall were total fermented beverages (respectively, 606 and 610 g/day; the main contributor was coffee), fermented dairy (respectively, 171 and 176 g/day; the main contributor was yoghurt), and fermented cereals/grains (respectively, 129 and 143 g/day; the main contributor was wholegrain bread). When expressed as a percentage of average daily energy intake, the main contributor changed for total fermented beverages to wine (respectively, 2.6 and 2.8%, for the FFQ and 24-h recall), and for total fermented dairy to cheese (respectively, 4.9 and 5.2%). For fermented cereals/grains, the main contributor remained wholegrain bread (respectively, 9.5 and 9.3%). Taking into account all fermented food groups, the mean percent (and absolute) difference between the FFQ and the 24-h recall data ranged from 0.3% (0.1 g/day) for cheeses to 10,224.4% (41.9 g/day) for buttermilk. Mean intakes were similar between the FFQ and 24-h recall methods for total fermented beverages (percent difference of − 0.7%) and in particular coffee (2.1%), fermented cereals and grains (− 9.7%), with smaller differences for specific assessments of brown bread, wholegrain bread, and rye breads, and total fermented dairy (− 2.8%), particularly for cheeses (− 0.3%). On the contrary, percent differences in mean intake for buttermilk, quark, and white bread were large (≥53%).

**Table 2:** Mean Intake of Fermented and Non-Fermented Products Assessed by FFQ and 24-Hour Recalls

	FFQ			24-h Recall			% Difference in group means (absolute difference)^b^
	Mean^a^	SD	Consumers	Mean^a^	SD	Consumers	
Energy, kcal/day	2143.7	504.8	809	2129.2	444.2	809	0.7 (14.5)
Fermented beverages	605.5 (4.5%)	376.7	782	609.6 (5.5%)	371.8	774	−0.7 (−4.1)
Coffee	457.6 (0.2%)	303.0	744	448.3 (0.8%)	287.9	742	2.1 (9.3)
Beer	82.8 (1.7%)	163.0	436	85.7 (1.8%)	174.4	267	−3.3 (−2.9)
Wine	65.0 (2.6%)	88.5	613	75.3 (2.8%)	100.0	507	−13.7 (−10.3)
Fermented cereals/grains	128.7 (15.0%)	52.1	806	142.6 (17.3%)	52.7	805	−9.7 (−13.9)
Brown bread	23.9 (2.6%)	36.0	535	24.6 (2.9%)	33.1	464	−2.6 (−0.7)
White bread	8.7 (1.1%)	15.6	531	18.6 (2.3%)	29.2	418	−53.4 (−9.9)
Wholegrain bread	82.4 (9.5%)	52.4	760	79.4 (9.3%)	55.5	723	3.8 (3)
Rye bread	3.3 (0.3%)	9.5	262	3.2 (0.3%)	11.7	103	5.1 (0.1)
Other bread	8.6 (1.1%)	14.3	274	7.7 (1.0%)	15.5	271	10.8 (0.9)
Pastries	1.8 (0.4%)	4.3	368	2.2 (0.4%)	6.7	117	−16.4 (−0.4)
Chocolate	5.6 (1.4%)	7.9	715	9.0 (2.2%)	10.8	575	−37.4 (−3.4)
Fermented dairy	170.6 (8.2%)	125.3	804	175.5 (8.5%)	129.8	795	−2.8 (−4.9)
Cheeses	31.6 (4.9%)	25.2	785	31.7 (5.2%)	20.4	761	−0.3 (−0.1)
Yoghurts	93.7 (2.5%)	90.5	704	81.8 (2.0%)	90.3	586	14.6 (11.9)
Quark	3.0 (0.1%)	16.5	60	13.9 (0.5%)	34.2	207	−78.6 (−10.9)
Buttermilk	42.3 (0.6%)	75.1	316	0.4 (0.01%)	7.3	3	10,224.4 (41.9)
Non-fermented dairy	152.7 (5.5%)	136.1	802	136.8 (5.5%)	132.4	752	11.7 (15.9)
Butter	3.0 (1.0%)	7.7	309	3.0 (1.0%)	6.0	308	0.8 (0)
Cream	3.0 (0.4%)	7.1	685	7.9 (0.7%)	13.4	444	−62.5 (−4.9)
Ice cream	6.1 (0.7%)	9.1	520	7.8 (0.9%)	14.7	242	−22.0 (−1.7)
Milk	140.7 (3.5%)	136.1	691	115.6 (2.6%)	131.9	591	21.7 (25.1)
Non-fermented soya	9.3 (0.3%)	34.3	683	11.1 (0.3%)	46.4	92	−15.9 (−1.8)

*FFQ* Food frequency questionnaire, *SD* Standard deviation

^a^Mean energy-adjusted intakes for the entire validation sample. Values are in g/day (and as % average daily energy intake) (*n =* 809)

^b^Percent difference is calculated using [(FFQ - Recall)/Recall] × 100% for each food or food group. For comparison, the absolute difference (FFQ – Recall) is also provided

High intake levels of non-fermented dairy foods were also observed in this population (recall 137 g/d and FFQ 153 g/day), the main contributor being milk (Table 2). While the percent difference in mean intakes was similar for butter (0.7%), a larger difference was observed for cream (− 62.5%) and non-fermented soya (− 15.9%).

Compared to group level percent differences in means, higher individual level percent differences in means was observed for total energy as well as multiple fermented food groups, with the most striking contrasts observed for beer, brown bread, white bread, rye bread, ‘other bread’, fermented dairy, cheeses, yoghurts, and buttermilk (Additional file 2, Table S2). Meanwhile, for wine, total fermented cereals/grains, quark, and ice cream, the mean percent differences on an individual level were improved. From the non-fermented food groups evaluated, milk and soya had large differences between percent differences in means determined on an individual compared to group level.

### Quintile cross-classification

The degree of potential misclassification of fermented foods was examined using quintile cross-classification (Table 3). All fermented food groups were characterised by over 50% of participants being classified into the same or adjacent quintile of intake, confirming good ranking ability (ranging from 57.8% for buttermilk to 88.5% for total fermented beverages). Furthermore, for total fermented beverages, coffee, and wine, almost 50% of participants were classified in the same quintile for both methods. While misclassification in the extreme quintiles was relatively low across the total fermented food groups (0.4–3.8%), a greater proportion of participants (5.3 to 6.8%) were grossly misclassified for some individual fermented foods including brown bread, ‘other bread’, pastries, quark, and buttermilk.

**Table 3:** Quintile Cross-Classification and Spearman’s Correlations for Fermented and Non-Fermented Foods

	Agreement of Quintiles for Food Group Intake^a^			Spearman’s Rank Correlation Coefficient (r_**s**_)	
Food Group	Same Quintile (%)	Adjacent Quintile (%)	Extreme Quintile (%)^b^	Crude	Energy-Adjusted
Fermented beverages	47.6	40.9	0.4	0.80**	0.78**
Coffee	48.8	37.2	0.9	0.76**	0.74**
Beer	39.9	35.0	1.7	0.67**	0.53**
Wine	46.4	38.9	0.3	0.76**	0.74**
Fermented cereals/grains	38.1	41.3	1.2	0.68**	0.63**
Brown bread	30.3	34.9	6.2	0.25**	0.28**
White bread	35.2	34.5	4.7	0.33**	0.35**
Wholegrain bread	38.8	37.9	1.9	0.61**	0.55**
Rye bread	33.9	34.2	3.0	0.43**	0.42**
Other bread	28.1	32.1	6.8	0.11**	0.17**
Pastries	29.2	33.7	5.4	0.20**	0.27**
Chocolate	27.8	39.8	3.8	0.36**	0.38**
Fermented dairy	43.1	40.2	0.9	0.68**	0.69**
Cheeses	32.9	38.1	2.2	0.46**	0.47**
Yoghurts	34.5	41.5	1.7	0.56**	0.55**
Quark	30.2	36.3	5.3	0.13**	0.31**
Buttermilk	25.2	32.6	5.6	0.10**	0.18**
Non-fermented dairy	40.9	41.5	0.9	0.68**	0.67**
Butter	36.6	37.6	2.5	0.48**	0.51**
Cream	28.7	30.5	5.4	0.20**	0.21**
Ice cream	26.1	35.1	5.7	0.23**	0.21**
Milk	40.9	41.9	1.1	0.67**	0.66**
Non-fermented soya	34.1	36.0	4.1	0.40**	0.41**

*FFQ* Food frequency questionnaire. **, *p* < 0.01.

^a^Mean energy-adjusted intake values for each food group were used to divide participants into quintiles

^b^Percentage of 1st quintile participants in the FFQ classified into the 5th quintile in the recall, or vice versa

Non-fermented dairy and soya food groups also had good agreement between dietary assessment tools in the quintile cross-classification, with over 50% of participants classified into the same or adjacent quintile of intake (Table 3). However, for cream and ice cream, a relatively higher percentage (5.4 and 5.7%, respectively) were misclassified into the extreme quintiles.

### Spearman’s correlations

Crude Spearman’s correlation coefficients ranged from to 0.10 (buttermilk) to 0.80 (fermented beverages) (Table 3). Energy-adjustment slightly increased the correlation coefficient for brown bread, white bread, ‘other bread’, pastries, chocolate, total fermented dairy, cheeses, quark, and buttermilk, and slightly decreased the correlation coefficient for other fermented food groups. Strong correlations (r_s_ ≥ 0.50) for both crude and energy-adjusted intakes were obtained for fermented beverages (including coffee, beer, wine), fermented cereals/grains (including wholegrain bread), and fermented dairy (including yoghurts). Correlation coefficients in the higher acceptable range were obtained for rye bread and cheeses (0.40 ≤ r_s_ ≤ 0.49), while correlation coefficients in the lower acceptable range were found for brown bread, white bread, pastries, and chocolate (0.20 ≤ r_s_ ≤ 0.39). Only for three fermented foods (‘other bread’, quark, buttermilk) was the correlation coefficient was less than 0.20. All correlations were statistically significant (*p* < 0.01). Crude and energy-adjusted attenuation factors for two 24-h recall replicates were consistently high for fermented beverages (0.81), coffee (0.85), beer (0.71), wine (0.64), while moderate values were observed in the range of attenuation factors for fermented cereals/grains (0.51), wholegrain bread (0.47), rye bread (0.54), fermented dairy (0.52), cheese (0.42), and yoghurts (0.53) (Additional file 3, Table S3). In comparison, lower values in the range of attenuation factors were obtained for brown bread (0.28), white bread (0.26), ‘other bread’ (0.19), pastries (0.23), chocolate (0.32), and quark (0.14). For buttermilk, accurate attenuation factors could not be calculated due to the low variance of the person-specific biases compared to the within- and between-person variances. As expected, for all food groups, attenuation factors improved with increasing replicates of the reference 24-h recall from two (0.14–0.86) to eight (0.24–1.0). Energy-adjustment had little effect on the attenuation factors. Sex-specific correlation coefficients (crude and energy-adjusted) were similar compared to those obtained for the total population, as well as between men and women, for virtually all fermented food groups (Additional file 6, Table S4). Energy-adjustment generally had a negligible effect on the sex-specific correlation coefficients, but were amplified for less commonly consumed foods, in the positive (i.e., other bread and buttermilk in men, quark and other bread in women) or negative direction (i.e., rye bread and quark in men, buttermilk in women).

Similarly, for non-fermented food groups, strong correlations were obtained for non-fermented dairy, including milk, while acceptable correlations were obtained for butter and non-fermented soya (in the higher range), and ice cream and cream (in the lower range) (Table 3). The crude and energy-adjusted attenuation factors obtained were high for non-fermented dairy (0.69–0.98) and butter (0.65–0.97), moderate for milk (0.57–0.72) and non-fermented soya (0.6–0.76), and lower for cream (0.32–0.5) and ice cream (0.18–0.63) (Additional file 3, Table S3). Sex-specific correlation coefficients (crude and energy-adjusted) for non-fermented food groups were similar compared to those obtained for the total population, as well as between men and women (Additional file 6, Table S4).

### Bland-Altman analyses

**Fig. 1:**
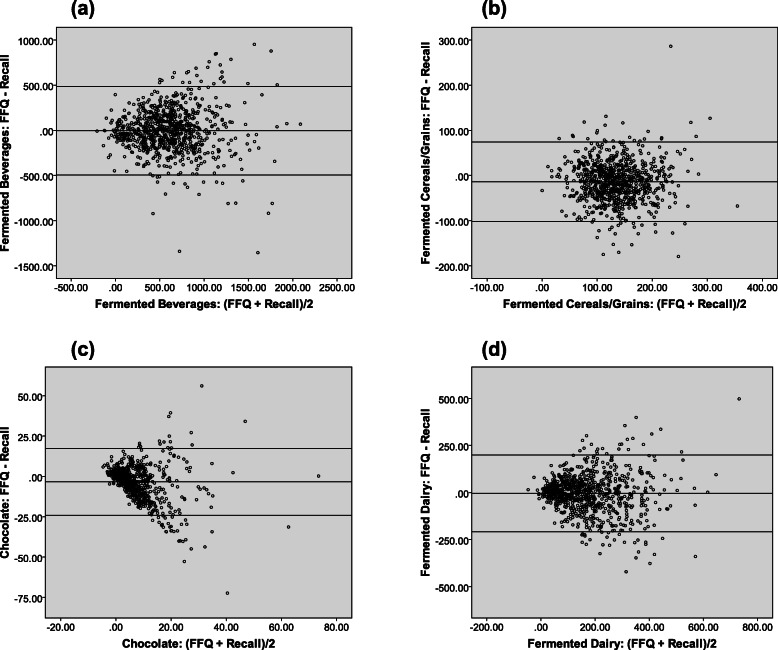
Bland-Altman plots demonstrating relative validity of FFQ versus 24-h recalls for main fermented food groups. Group-level relative validity assessed for: (**a**) fermented beverages, (**b**) fermented cereals/grains, (**c**) chocolate, and (**d**) fermented dairy. The middle line indicates the mean difference, while the upper and lower lines indicate the 95% confidence intervals, respectively [calculated as: mean ± (standard deviation of the mean difference X 1.96)]

The results of the Bland-Altman analyses revealed good agreement in group-level intakes for total fermented beverages, including coffee and beer (Additional file 2, Table S2). Good agreement was also demonstrated for brown bread, wholegrain bread, rye bread, ‘other bread’, pastries, chocolate, fermented dairy, and cheeses (mean difference between − 0.1 to 9.3 g/day; p_difference_ ≥ 0.05). However, for wine, total fermented cereals/grains, white bread, chocolate, yoghurts, quark, and buttermilk, significant differences were found between the two dietary assessment methods (mean difference between − 3.4 to 41.9, p_difference_ < 0.0001). The results of the regression analyses further revealed a significant amount of proportional bias for wine, white bread, rye bread, pastries, chocolate, cheese, quark, and buttermilk (p_slope_ < 0.0001). For certain foods (wine, white bread, pastries, chocolate, cheese, and quark), the FFQ tended to consistently underestimate their consumption compared to the 24-h recalls, while intakes were overestimated for others (rye bread and buttermilk). A small bias for coffee, beer, brown bread, and ‘other bread’ was also observed (*p* < 0.05) (Additional file 2, Table S2). These results were also confirmed visually in the Bland-Altman plots for the main fermented food groups (Fig. 1) and subgroups (Additional file 4, Figure S1).

For non-fermented foods, good agreement between dietary assessment methods was demonstrated for butter and non-fermented soya (mean difference between 0.02 to − 1.8; *p* ≥ 0.05), while poor agreement was revealed for total non-fermented dairy, cream, ice cream, and milk (mean difference between − 4.9 to 25.1; p_difference_ < 0.0001) (Additional file 2, Table S2). The results of the regression analyses demonstrated significant proportional bias for butter, cream, ice cream, and non-fermented soya (p_slope_ < 0.0001) (Table 1 and Additional file 5, Figure S2).

### Summary of validity assessment

A summary assessment of the different aspects of validity between the FFQ and 24-h recall methods is provided in Table 4.

**Table 4:** Summary of Validity Assessments Between the FFQ and 24-h Recalls for Fermented and Non-Fermented Foods

Method	% Difference	Correlation Coefficient	Quintile Cross-Classification	Bland-Altman^**a**^
Aspect of Validity Measured	Agreement (group level)	Strength and direction of association (individual level)	Agreement (individual level)	Presence, direction, and extent of bias (group level)
Interpretation Criteria	**Acceptable**: 0.0 to 10.0% **Poor**: > 10%	**Good**: ≥0.50 **Acceptable**: 0.20 to 0.39 (lower range); 0.40 to 0.49 (higher range) **Poor**: < 0.20	**Good**: ≥50% in same/adjacent quintile; ≤5% in extreme quintile **Poor**: < 50% in same/adjacent quintile; > 5% in extreme quintile	**Good**: ***p*** > 0.05 **Poor**: ***p*** ≤ 0.05
Fermented beverages	**Acceptable**	**Good**	**Good**	**Good**
Coffee	**Acceptable**	**Good**	**Good**	**Good (bias)**
Beer	Poor	**Good**	**Good**	**Good (bias)**
Wine	Poor	**Good**	**Good**	Poor (bias)
Fermented cereals/grains	**Acceptable**	**Good**	**Good**	Poor
Brown bread	**Acceptable**	**Acceptable (low)**	Poor	**Good (bias)**
White bread	Poor	**Acceptable (low)**	**Good**	Poor (bias)
Wholegrain bread	**Acceptable**	**Good**	**Good**	**Good**
Rye bread	**Acceptable**	**Acceptable (high)**	**Good**	**Good (bias)**
Other bread	Poor	Poor	Poor	**Good (bias)**
Pastries	Poor	**Acceptable (low)**	Poor	**Good (bias)**
Chocolate	Poor	**Acceptable (low)**	**Good**	Poor (bias)
Fermented dairy	**Acceptable**	**Good**	**Good**	**Good**
Cheese	**Acceptable**	**Acceptable (high)**	**Good**	**Good (bias)**
Yoghurts	Poor	**Good**	**Good**	Poor
Quark	Poor	Poor	Poor	Poor (bias)
Buttermilk	Poor	Poor	Poor	Poor (bias)
Non-fermented dairy	Poor	**Good**	**Good**	Poor
Butter	**Acceptable**	**Acceptable (high)**	**Good**	**Good (bias)**
Cream	Poor	**Acceptable (low)**	Poor	Poor (bias)
Ice cream	Poor	**Acceptable (low)**	Poor	Poor (bias)
Milk	Poor	**Good**	**Good**	Poor
Non-fermented soya	Poor	**Acceptable (high)**	**Good**	**Good (bias)**

*FFQ* Food frequency questionnaire. Acceptable and good validity assessment outcomes are bolded.

^a^The presence of proportional bias for each food group is indicated in brackets

## Discussion

### Consumption of fermented foods by adults in the Netherlands

While it has been previously estimated that 5 to 40% of foods in the human diet are fermented [39], a quantitative evaluation of the contribution of fermented foods to the human diet had not been conducted prior to this report. Based on the present analysis, approximately 16 to 18% of foods consumed in this population are fermented food items, while a further 9 to 14% are composite dishes that contain a fermented ingredient, indicating that there is a high prevalence of fermented foods in the Dutch diet. These estimates are also likely to be valid for other countries (in Europe or worldwide) in which primarily Western diets are consumed.

### Reliability of the current FFQ for estimating fermented food intake

In the present study, we also assessed the relative validity of a FFQ compared to multiple 24-h recalls for estimating the intake of fermented foods in a Dutch adult population. Using a combination of validation methods, including percent difference, quintile cross-classification, Spearman’s correlation, and Bland-Altman plots, fermented food groups that had acceptable or good validity across all measures included total fermented beverages, coffee, wholegrain bread, rye bread, fermented dairy, and cheese. From the non-fermented food groups that were assessed, butter was the only food with uniformly good/acceptable validity. In addition, wine, beer, fermented cereals/grains, white bread, chocolate, yoghurts, non-fermented dairy, milk, and non-fermented soya all had good ranking ability (as indicated by the strong correlation coefficients and high agreement in quintile cross-classification), albeit poor parametric assessment of differences (as indicated by the low agreement in percent difference and Bland-Altman).

Fermented foods with the highest consumption levels included coffee (~ 453 g/day; ~ 0.5% of daily energy intake), yoghurts (~ 88 g/day; ~ 2.3% of daily energy intake), beer (~ 84 g/day; ~ 1.8% of daily energy intake), wholegrain bread (~ 81 g/day; ~ 9.4% of daily energy intake), wine (~ 65 g/day; ~ 2.7% of daily energy intake), and cheese (~ 32 g/day; ~ 5.1% of daily energy intake). These foods, with the exception of coffee, also correspond to the top fermented foods contributing to total daily energy intake in this study. Comparing our findings to studies in other European populations, mean daily intakes were similar for coffee (404 g/day), bread products (64 to 146 g/day), butter (5 g/day), cheese (25 to 58 g/day), yoghurt (95 g/day), and soya products (6 to 10 g/day) [12, 13, 15, 40]; however, milk consumption in our study is a little lower than previously reported (220 to 230 g/day) [12, 13, 15]. As indicated previously, the comparison of mean energy-adjusted daily intakes for the fermented food groups revealed group-level differences ranging from 0.3% (for cheeses) to 10,224% (for buttermilk). Foods that are consumed by the majority of the population on a regular basis, such as coffee, bread, and cheeses, showed comparable intakes for 24-recall and FFQ assessments (− 0.3 to 9.7%), which was expected. The most striking differences in mean intakes were for buttermilk and quark (10,224 and − 78.6%, respectively), which might be a consequence of the difference in number of consumers between the FFQ and recall for these foods (60 vs. 207 for quark, 316 vs. 3 for buttermilk). Moreover, the results of both comparison of mean intakes and Bland-Altman revealed that intakes for most fermented food groups were slightly underestimated by the FFQ when compared to the 24-h recalls, which is expected since the 24-h recall, by design, generally captures a greater proportion of the diet than the FFQ.

Since a critical measure of success for an FFQ is its ability to accurately rank individuals into high- and low-intakes based on their habitual diet [13], we evaluated ranking ability using both Spearman’s correlation and quintile cross-classification. High Spearman’s correlation coefficients (r_s_ ≥ 0.50) were obtained for all fermented beverage groups (total fermented beverages, coffee, beer, wine), total fermented cereals/grains, wholegrain bread, total fermented dairy, yoghurts, total non-fermented dairy, and milk. Since the use of different dietary assessment instruments in distinct populations could affect results, we compared our results with those obtained from other studies for similar food groups. Streppel et al. [15] assessed the relative validity of a previous version of the FFQ used in the current study in 128 elderly Dutch individuals. Comparing the FFQ data to three 24-h recalls, Pearson’s correlations of 0.71 to 0.93 for bread, 0.46 to 0.61 for cheese, 0.68 to 0.75 for milk and milk products, and 0.50 to 0.66 for soya and vegetarian products were obtained. Similar validation studies have been conducted in 161 German adults [12], 100 Belgian adults [14], 1213 German adolescents [13], and 56 Swiss adults [40], comparing a FFQ to multiple 24-h recalls, 7-day estimated diet records, diet history interviews, and 4-day weighted food records, respectively. Collectively, the correlations obtained in these studies of 0.69 to 0.78 for coffee, 0.40 to 0.42 for dairy products, 0.63 for butter, 0.63 to 0.66 for milk, 0.49 for curd cheese, soured milk, and yoghurt, 0.25 to 0.61 for cheese, 0.59 for ice cream, and 0.16 to 0.48 for bread and cereals, are similar to those determined in the current study (0.78 to 0.80 for coffee, 0.20 to 0.21 for butter, 0.66 to 0.67 for milk, 0.46 to 0.47 for cheese, 0.55 to 0.56 for yoghurt, 0.21 to 0.23 for ice cream, and 0.63 to 0.68 for total fermented cereals/grains). The interesting exception is that the correlation coefficients determined for total fermented cereals/grains (r_s_ 0.63) and wholegrain bread (r_s_ 0.55) in our study are slightly lower than those reported in Streppel et al. [15] (r_s_ 0.71 to 0.93 for bread). Although total bread consumption in this elderly population (126 to 133 g/day) is comparable to total fermented cereals/grains intake by the study cohort described in the current publication (129 to 143 g/day), older adults tend to have more stable diets and different dietary patterns than those of younger adults, which might account for this difference in correlation coefficients. The attenuation factors obtained for the food groups investigated in this study were considerably higher for fermented beverages, coffee, beer, and wine (range between 0.64–1.0) than for brown bread, white bread, ‘other bread’, pastries, chocolate, and quark (range between 0.14–0.77). Since attenuation factors closer to one indicates a better overall estimation of intake [36], these results suggest that intake estimates for all fermented beverages are reliable across the dietary methods used here. Meanwhile, intake estimates were weaker for fermented cereals and grains, wholegrain bread, rye bread, fermented dairy, cheeses, and yoghurts, and weakest for brown bread, white bread, ‘other bread’, pastries, chocolate, and quark. These effects correspond with other assessments of validity for the food groups investigated.

Our results for quintile cross-classification further supported a high level of agreement between the FFQ and 24-h recall in ranking participants for the majority of fermented foods, non-fermented dairy, and soya, even when using more stringent criteria of quintiles and cut-offs of 5% for misclassification. Other validation studies have reported similar cross-classifications for cheese (46.8% same quartile, 50% same tertile), milk (45.6–49.5% same quartile), milk and soya products (57.4% same tertile), butter (49.7–50.3% same quartile) [12–14], and slightly lower cross-classification for coffee (75.2% same quartile) and ice cream (44.1% same quartile) [13]. Interestingly, in these studies and ours, bread products had a lower accurate classification rate (33.1–39.2% in same tertile or quartile, and 6.4–8.4% in opposite quartile [12–14]; 28.1–38.8% in same quintile and 1.2–6.8% in opposite quintile (present study)). Although total fermented cereals/grains had good cross-classification, the same is not true for the corresponding subgroups, which may be also be attributed to the misclassification of bread products by consumers for FFQs. Sporadically consumed foods (e.g., quark) or seasonal foods (e.g., ice cream) also tended to have a higher discordance and degree of misclassification between methods. However, these results were expected due to the lower probability of assessing such foods on recall days, compared to the FFQ which evaluates a larger reference period [12].

### Study strengths and limitations

A strength of this study is the inclusion of a large population size for validation. Moreover, we utilised multiple assessment methods, which allows for a more comprehensive evaluation of relative validity at both the individual and group level [31]. Notwithstanding, there are several limitations to address, the most dominant of which relate to the inherent limitations associated with using the FFQ and 24-h recall to measure dietary intake. Firstly, in an ideal validation, the comparison and reference methods should have independent error sources [9]. Both the FFQ and 24-h recall rely on the memories and perceptions of the participants, which can lead to higher estimates of validity [13]. Moreover, both methods rely on the same food composition table and tools to classify foods and quantify portion sizes, as an additional source of correlated error. Thus, only relative validity could be determined in this study. Secondly, estimates of portion sizes were performed using standard portion sizes and household measures. While these are commonly used approaches, different interpretations of household portion sizes can lead to misclassification and measurement bias. In particular, this misclassification may be more pronounced in estimates of absolute intake (i.e., in g/day), while a small effect is anticipated on the ranking ability of the FFQ (i.e., into quintiles of intake). Ongoing innovations in technology-based tools are aimed to reduce this source of measurement bias in dietary assessment. Thirdly, foods that are not frequently consumed or consumed only seasonally may be inconsistently or unevenly captured, depending on when the diet was assessed. Fourthly, since the dietary assessments in NQplus were performed from 2011 to 2014, we cannot exclude the possibility of changes in diet that would obscure the underlying assumption that participants have very stable diets at the time of assessment.

A further aspect worth highlighting is that the FFQ used in the NQplus study was not specifically designed to assess the intake of fermented foods; consequently, not all fermented food products could be captured by this method and some may only be represented by a food group comprising fermented and non-fermented foods. Indeed, 247 fermented foods were reported for the 24-h recall compared to only 39 in the FFQ. The fermented foods in this validation study primarily consisted of foods common to the Western diet (e.g., coffee, breads, dairy), but there exists a wealth of fermented foods from other cultures and regions that are increasingly consumed due to globalisation (e.g., kombucha, kefir, kimchi, natto, tempe). Novel fermented foods are also being developed, driven by the realisation of their potential impacts on health [41], and that consumption of fermented foods with live microorganisms may promote a healthy gut microbiota [42, 43]. Conversely, some traditionally fermented products (e.g., pickles, olives, mozzarella) are no longer fermented due to modernisation of food processing technologies [44]. As such, fermented and non-fermented versions of the same products become indistinguishable to consumers. This emphasises the importance for developing a FFQ specific for fermented food products. Nevertheless, the present study reveals the strengths and limitations of each tool for assessing fermented food intake, highlights the need to design a FFQ to specifically assess fermented food intake, and aids in our goal of developing unbiased biomarkers of intake for fermented foods. Improved dietary assessment of fermented foods is expected to aid future trials that investigate associations between fermented food intake and (cardiometabolic) health.

## Conclusions

About a fifth of the Dutch diet consists of fermented food items. Adequate to good relative validity was determined for the FFQ compared to the 24-h recall across all statistical measures for commonly consumed foods, including total fermented beverages, coffee, wholegrain bread, rye bread, total fermented dairy, cheeses, as well as butter (non-fermented). For wine, beer, total fermented cereals/grains, white bread, chocolate, yoghurts, as well as total non-fermented dairy, milk, and non-fermented soya, good ranking ability of participants into their levels of consumption could be established, albeit poor agreement in absolute intakes. For quark and buttermilk (fermented), as well as cream and ice cream (non-fermented), acceptable relative validity between the two methods could not be established; thus, the intakes for these food groups should be interpreted with caution in future studies using this population. Developing a FFQ specific for fermented foods would be valuable to capture global fermented foods that are increasingly consumed, and to delineate between fermented and non-fermented versions of foods that could obscure investigations between fermented food intake and health.

## Supplementary Information

**Additional file 1:** **Table S1.** Identification and Classification of Fermented Foods from the FFQ and 24-h Recalls in NQplus.

**Additional file 2:** **Table S2.** Mean Individual Percent Difference and Test Statistics from Bland-Altman Analyses for the Validation Sample (*n* = 809).

**Additional file 3:** **Table S3.** Attenuation Factors for the Reference 24-h Recall Compared to the Food Frequency Questionnaire.

**Additional file 4:** **Figure S1.** Bland-Altman plots demonstrating relative validity of the FFQ versus 24-h recalls for fermented food subgroups.

**Additional file 5:** **Figure S2.** Bland-Altman plots demonstrating relative validity of the FFQ versus 24-h recalls for non-fermented foods.

**Additional file 6:** **Table S4.** Sex-Specific Spearman’s Correlations for Fermented and Non-Fermented Foods.

## Data Availability

The datasets supporting the conclusions of this article are included within the article (and its additional files).

## Abbreviations

24-h: 24-hour
DNFCS: Dutch National Food Consumption Survey
FFQ: Food frequency questionnaire
NDARD: National Dietary Assessment Reference Database
NQplus: Nutrition Questionnaires plus
