# Supplementary material for: Prevalence of fermented foods in the Dutch adult diet and validation of a food frequency questionnaire for estimating their intake in the NQplus cohort
Source: BMC Nutr. 2020 Dec 3;6:69. doi: 10.1186/s40795-020-00394-z (PMC7712622; doi:10.1186/s40795-020-00394-z)
Supplement: Supplementary file 1 — Additional file 1: Table S1. Identification and Classification of Fermented Foods from the FFQ and 24-h Recalls in NQplus. [file 40795_2020_394_MOESM1_ESM.pdf]

| Table S1 Identification and Classification of Fermented Foods from the FFQ and 24-h Recalls in NQplus[a] | | | | | |
|---|---|---|---|---|---|
| **Food[b]** | **Fermentation Status** | **Food Group** | **Subgroup** | **(Main) Fermentation Organism** | **Mean intakes (g/day)** |
| **Food Frequency Questionnaire** | | | | | |
| Beer | Fermented | Beverages | Beer | Yeast | 83.69 |
| Low -alcohol beer | Fermented | Beverages | Beer | Yeast | 3.92 |
| Coffee | Fermented | Beverages | Coffee | Endogenous microorganisms | 452.67 |
| Wine, other | Fermented | Beverages | Wine | Endogenous microorganisms | 19.24 |
| Wine, red | Fermented | Beverages | Wine | Endogenous microorganisms | 46.29 |
| Brow n bread | Fermented | Cereals/grains | Bread, brow n | Yeast | 3.18 |
| Brow n bread (sliced) | Fermented | Cereals/grains | Bread, brow n | Yeast | 21.54 |
| Bread w ith raisins | Fermented | Cereals/grains | Bread, other | Yeast | 8.63 |
| Rye bread | Fermented | Cereals/grains | Bread, rye | Yeast | 2.91 |
| Biscuit (bread), w hite | Fermented | Cereals/grains | Bread, w hite | Yeast | 0.69 |
| White bread | Fermented | Cereals/grains | Bread, w hite | Yeast | 3.35 |
| White bread (sliced) | Fermented | Cereals/grains | Bread, w hite | Yeast | 4.57 |
| Biscuit (bread), w hole w heat | Fermented | Cereals/grains | Bread, w holegrain | Yeast | 1.69 |
| Multiple grain bread | Fermented | Cereals/grains | Bread, w holegrain | Yeast | 2.66 |
| Multiple grain bread (sliced) | Fermented | Cereals/grains | Bread, w holegrain | Yeast | 29.74 |
| Whole w heat bread (sliced) | Fermented | Cereals/grains | Bread, w holegrain | Yeast | 46.89 |
| Croissant | Fermented | Cereals/grains | Pastry | Yeast | 1.85 |
| Milk chocolate | Fermented | Cocoa-based products | Chocolate | Endogenous microorganisms | 1.94 |
| Pure chocolate | Fermented | Cocoa-based products | Chocolate | Endogenous microorganisms | 2.94 |
| White chocolate | Fermented | Cocoa-based products | Chocolate | Endogenous microorganisms | 0.53 |
| Buttermilk | Fermented | Dairy | Buttermilk | Bacteria | 43.46 |

| | | | | | |
|---|---|---|---|---|---|
| Low -fat cheese (20+/30+) | Fermented | Dairy | Cheese, hard | Bacteria | 5.12 |
| Regular cheese (40+) | Fermented | Dairy | Cheese, hard | Bacteria | 1.67 |
| Regular cheese (48+) | Fermented | Dairy | Cheese, hard | Bacteria | 12.47 |
| Cheese as snack | Fermented | Dairy | Cheese, unspecified | Bacteria | 3.23 |
| Cheese w ith hot meal | Fermented | Dairy | Cheese, unspecified | Bacteria | 3.08 |
| Fat luxury cheese | Fermented | Dairy | Cheese, unspecified | Bacteria | 1.86 |
| Less-fat luxury cheese | Fermented | Dairy | Cheese, unspecified | Bacteria | 1.99 |
| Unknow n cheese | Fermented | Dairy | Cheese, unspecified | Bacteria | 0.58 |
| (Fruit) quark w ith breakfast | Fermented | Dairy | Quark | Bacteria | 3.43 |
| Full (fruit) yoghurt | Fermented | Dairy | Yoghurt | Bacteria | 4.45 |
| Full yoghurt | Fermented | Dairy | Yoghurt | Bacteria | 12.08 |
| Semi-skim (fruit) yoghurt | Fermented | Dairy | Yoghurt | Bacteria | 5.73 |
| Semi-skim yoghurt | Fermented | Dairy | Yoghurt | Bacteria | 24.75 |
| Skim (fruit) yoghurt | Fermented | Dairy | Yoghurt | Bacteria | 13.51 |
| Skim yoghurt | Fermented | Dairy | Yoghurt | Bacteria | 31.93 |
| Unknow n yoghurt | Fermented | Dairy | Yoghurt | Bacteria | 3.15 |
| Marmite | Fermented | Other | Marmite | Yeast | 0.02 |
| Dressing of oil/vinegar | Fermented | Other | Vinegar | Bacteria/yeast | 1.34 |
| Sandw ich sausage | Unclear if sausage is fermented. | NA | NA | NA | 3.05 |
| Chocolate spread | Composite dish/contains a fermented ingredient but is not itself fermented. | NA | NA | NA | 4.92 |
| Breakfast drink | Composite dish/contains a fermented ingredient but is not itself fermented. | NA | NA | NA | 3.28 |
| Mousse (dessert) | Composite dish/contains a fermented ingredient but is not itself fermented. | NA | NA | NA | 5.28 |
| Pizza | Composite dish/contains a fermented ingredient but is not itself fermented. | NA | NA | NA | 12.37 |
| Dressing of light mayonnaise | Composite dish/contains a fermented ingredient but is not itself fermented. | NA | NA | NA | 1.68 |
| Dressing of mayonnaise | Composite dish/contains a fermented ingredient but is not itself fermented. | NA | NA | NA | 0.25 |

| | | | | | |
|---|---|---|---|---|---|
| Mayonnaise | Composite dish/contains a fermented ingredient but is not itself fermented. | NA | NA | NA | 0.85 |
| Light mayonnaise | Composite dish/contains a fermented ingredient but is not itself fermented. | NA | NA | NA | 1.19 |
| Ketchup | Composite dish/contains a fermented ingredient but is not itself fermented. | NA | NA | NA | 1.44 |
| Nutrition biscuit | Unclear if yeast is used in the cooking process. | NA | NA | NA | 4.05 |
| Mayonnaise with snack | Composite dish/contains a fermented ingredient but is not itself fermented. | NA | NA | NA | 0.16 |
| Light mayonnaise with snack | Composite dish/contains a fermented ingredient but is not itself fermented. | NA | NA | NA | 0.17 |
| Ketchup with snack | Composite dish/contains a fermented ingredient but is not itself fermented. | NA | NA | NA | 0.28 |
| Eggnog | Composite dish/contains a fermented ingredient but is not itself fermented. | NA | NA | NA | 0.20 |
| Strong drink | Unclear if this is an alcoholic drink that is produced by fermentation. | NA | NA | NA | 4.01 |
| Skim milk or buttermilk | Unclear category (will check how highly reported and intake level). | NA | NA | NA | 2.08 |
| Whole wheat cracker | Unclear if yeast is used in the cooking process. | NA | NA | NA | 1.88 |
| Liver sausage | Unclear if sausage is fermented. | NA | NA | NA | 0.42 |
| **24-h Recall** | | | | | |
| Beer, >7 vol% alcohol | Fermented | Beverages | Beer | Yeast | 382.25 |
| Beer, alcohol free <0.1 vol% alcohol | Fermented | Beverages | Beer | Yeast | 341.49 |
| Beer, brown | Fermented | Beverages | Beer | Yeast | 388.57 |
| Beer, low alcohol 0.1-1.2 vol% alcohol | Fermented | Beverages | Beer | Yeast | 250.00 |
| Beer, pilsner (pale lager) | Fermented | Beverages | Beer | Yeast | 464.51 |
| Beer with fruit flavour | Fermented | Beverages | Beer | Yeast | 330.00 |
| Cider | Fermented | Beverages | Cider | Yeast | 211.67 |
| Cappuccino, freshly made | Fermented | Beverages | Coffee | Endogenous microorganisms | 199.67 |

| | | | | | |
|---|---|---|---|---|---|
| Coffee, prepared | Fermented | Beverages | Coffee | Endogenous microorganisms | 209.08 |
| Coffee, cappuccino instant prepared | Fermented | Beverages | Coffee, instant | Endogenous microorganisms | 236.56 |
| Coffee with milk from vending machine | Fermented | Beverages | Coffee, instant | Endogenous microorganisms | 302.27 |
| Coffee with sugar and milk from vending machine | Fermented | Beverages | Coffee, instant | Endogenous microorganisms | 222.79 |
| Madeira | Fermented | Beverages | Wine | Endogenous microorganisms | 35.67 |
| Port wine | Fermented | Beverages | Wine | Endogenous microorganisms | 74.28 |
| Sherry | Fermented | Beverages | Wine | Endogenous microorganisms | 81.66 |
| Medicinal wine (pleegzusterbloedwijn) | Fermented | Beverages | Wine | Endogenous microorganisms | 75.00 |
| Wine, red | Fermented | Beverages | Wine | Endogenous microorganisms | 151.68 |
| Wine, rose | Fermented | Beverages | Wine | Endogenous microorganisms | 157.12 |
| Wine, white dry | Fermented | Beverages | Wine | Endogenous microorganisms | 151.79 |
| Wine, white sweet | Fermented | Beverages | Wine | Endogenous microorganisms | 129.42 |
| Baguette, brown | Fermented | Cereals/grains | Bread, brown | Yeast | 65.52 |
| Bread (Blue Brand Goede Start), light brown | Fermented | Cereals/grains | Bread, brown | Yeast | 70.00 |
| Bread brown with pumpkin seeds | Fermented | Cereals/grains | Bread, brown | Yeast | 78.57 |
| Bread brown with seeds | Fermented | Cereals/grains | Bread, brown | Yeast | 71.27 |
| Bread brown with sunflower seeds | Fermented | Cereals/grains | Bread, brown | Yeast | 71.26 |
| Bread brown wheat | Fermented | Cereals/grains | Bread, brown | Yeast | 70.76 |
| Bread brown/wholemeal with muesli | Fermented | Cereals/grains | Bread, brown | Yeast | 51.89 |
| Bread brown/wholemeal with pumpkin seeds | Fermented | Cereals/grains | Bread, brown | Yeast | 61.76 |

| | | | | | |
|---|---|---|---|---|---|
| Bread brow n/w holemeal w ith sunflow er seeds | Fermented | Cereals/grains | Bread, brow n | Yeast | 73.04 |
| Roll brow n, hard | Fermented | Cereals/grains | Bread, brow n | Yeast | 68.01 |
| Roll brow n, soft | Fermented | Cereals/grains | Bread, brow n | Yeast | 76.25 |
| Baguette w ith cheese-onion | Fermented | Cereals/grains | Bread, other | Yeast | 79.00 |
| Bread, ciabatta, no filling | Fermented | Cereals/grains | Bread, other | Yeast | 87.65 |
| Bread, currant | Fermented | Cereals/grains | Bread, other | Yeast | 49.83 |
| Bread, currant w ith almond paste | Fermented | Cereals/grains | Bread, other | Yeast | 47.03 |
| Bread, linseed | Fermented | Cereals/grains | Bread, other | Yeast | 48.25 |
| Bread loaf, gluten-free, Glutafin | Fermented | Cereals/grains | Bread, other | Yeast | 78.11 |
| Bread, low in carbohydrates | Fermented | Cereals/grains | Bread, other | Yeast | 53.45 |
| Bread, raisin | Fermented | Cereals/grains | Bread, other | Yeast | 52.47 |
| Bread, raisin/current average | Fermented | Cereals/grains | Bread, other | Yeast | 48.81 |
| Bread ,w heat malt Tarvo | Fermented | Cereals/grains | Bread, other | Yeast | 79.80 |
| Bun, currant/raisin | Fermented | Cereals/grains | Bread, other | Yeast | 58.21 |
| Bun w ith vanilla custard | Fermented | Cereals/grains | Bread, other | Yeast | 76.67 |
| Coconut bread, sw eetened, sliced | Fermented | Cereals/grains | Bread, other | Yeast | 10.71 |
| Stollen w ith almond/paste, average | Fermented | Cereals/grains | Bread, other | Yeast | 73.31 |
| Stollen w ith almond/paste w ith nuts | Fermented | Cereals/grains | Bread, other | Yeast | 62.56 |
| Stollen w ith almond/paste w ithout nuts | Fermented | Cereals/grains | Bread, other | Yeast | 56.00 |
| Bread, rye, average | Fermented | Cereals/grains | Bread, rye | Yeast | 53.37 |
| Bread, rye, dark | Fermented | Cereals/grains | Bread, rye | Yeast | 46.31 |
| Bread, rye, light | Fermented | Cereals/grains | Bread, rye | Yeast | 72.77 |
| Bread crumbs | Fermented | Cereals/grains | Bread, toasted | Yeast | 9.11 |
| Crackers cream | Fermented | Cereals/grains | Bread, toasted | Yeast | 15.08 |
| Crackers cream, low sodium | Fermented | Cereals/grains | Bread, toasted | Yeast | 26.22 |
| Crispbakes, Dutch | Fermented | Cereals/grains | Bread, toasted | Yeast | 12.19 |
| Crispbakes, Dutch w holemeal | Fermented | Cereals/grains | Bread, toasted | Yeast | 13.42 |
| Crispbread, average | Fermented | Cereals/grains | Bread, toasted | Yeast | 18.05 |

| | | | | | |
|---|---|---|---|---|---|
| Crispbread, Cracottes | Fermented | Cereals/grains | Bread, toasted | Yeast | 13.08 |
| Crispbread, Cracottes Vital | Fermented | Cereals/grains | Bread, toasted | Yeast | 22.50 |
| Crispbread, gold-brow n | Fermented | Cereals/grains | Bread, toasted | Yeast | 17.74 |
| Crispbread, high fibre | Fermented | Cereals/grains | Bread, toasted | Yeast | 17.63 |
| Crispbread, light | Fermented | Cereals/grains | Bread, toasted | Yeast | 9.35 |
| Crispbread, poppy seeds | Fermented | Cereals/grains | Bread, toasted | Yeast | 30.00 |
| Crispbread, sandw ich Wasa | Fermented | Cereals/grains | Bread, toasted | Yeast | 31.67 |
| Crispbread, sesame | Fermented | Cereals/grains | Bread, toasted | Yeast | 20.05 |
| Crispbread, w holemeal | Fermented | Cereals/grains | Bread, toasted | Yeast | 21.10 |
| Crispbread, w holemeal Cracottes | Fermented | Cereals/grains | Bread, toasted | Yeast | 24.71 |
| Pretzel sticks | Fermented | Cereals/grains | Bread, toasted | Yeast | 18.76 |
| Toast | Fermented | Cereals/grains | Bread, toasted | Yeast | 12.19 |
| Toast, Melba natural | Fermented | Cereals/grains | Bread, toasted | Yeast | 10.72 |
| Toast, Melba other varieties | Fermented | Cereals/grains | Bread, toasted | Yeast | 21.08 |
| Baguette, w hite | Fermented | Cereals/grains | Bread, w hite | Yeast | 68.90 |
| Bread, Blue Band Goede Start w hite bread | Fermented | Cereals/grains | Bread, w hite | Yeast | 34.17 |
| Bread, pita w hite | Fermented | Cereals/grains | Bread, w hite | Yeast | 89.48 |
| Bread, Tijger w hite | Fermented | Cereals/grains | Bread, w hite | Yeast | 73.16 |
| Bread, w hite average milk/w ater based | Fermented | Cereals/grains | Bread, w hite | Yeast | 57.64 |
| Bread, w hite milk based | Fermented | Cereals/grains | Bread, w hite | Yeast | 58.29 |
| Bread, w hite Turkish | Fermented | Cereals/grains | Bread, w hite | Yeast | 99.50 |
| Bread, w hite w ith sugar Suikerbrood | Fermented | Cereals/grains | Bread, w hite | Yeast | 57.81 |
| Bread, w hite w ith sunflow er seeds | Fermented | Cereals/grains | Bread, w hite | Yeast | 74.25 |
| Bread, w hite, w ater based | Fermented | Cereals/grains | Bread, w hite | Yeast | 62.67 |
| Roll, w hite, hard | Fermented | Cereals/grains | Bread, w hite | Yeast | 76.06 |
| Roll, w hite, soft | Fermented | Cereals/grains | Bread, w hite | Yeast | 62.19 |
| Bread, current, w holemeal | Fermented | Cereals/grains | Bread, w holegrain or w holemeal | Yeast | 53.81 |

| | | | | | |
|---|---|---|---|---|---|
| Bread, multigrain, average with seeds | Fermented | Cereals/grains | Bread, wholegrain or wholemeal | Yeast | 72.70 |
| Bread, multigrain flour with seeds | Fermented | Cereals/grains | Bread, wholegrain or wholemeal | Yeast | 91.33 |
| Bread, multigrain, wholemeal with Becel | Fermented | Cereals/grains | Bread, wholegrain or wholemeal | Yeast | 67.63 |
| Bread, multigrain, wholemeal with seeds | Fermented | Cereals/grains | Bread, wholegrain or wholemeal | Yeast | 73.21 |
| Bread, sourdough, wholemeal | Fermented | Cereals/grains | Bread, wholegrain or wholemeal | Yeast/bacteria | 81.48 |
| Bread, Tijger brown, wheat | Fermented | Cereals/grains | Bread, wholegrain or wholemeal | Yeast | 85.37 |
| Bread, wheat rye, wholemeal | Fermented | Cereals/grains | Bread, wholegrain or wholemeal | Yeast | 70.00 |
| Bread, wholemeal, average | Fermented | Cereals/grains | Bread, wholegrain or wholemeal | Yeast | 70.38 |
| Bread, wholemeal, fine | Fermented | Cereals/grains | Bread, wholegrain or wholemeal | Yeast | 74.45 |
| Bread, wholemeal with nuts | Fermented | Cereals/grains | Bread, wholegrain or wholemeal | Yeast | 61.96 |
| Bread, wholemeal with pumpkin seeds | Fermented | Cereals/grains | Bread, wholegrain or wholemeal | Yeast | 66.67 |
| Bread wholemeal with seeds | Fermented | Cereals/grains | Bread, wholegrain or wholemeal | Yeast | 74.28 |
| Bread wholemeal with sunflower seeds | Fermented | Cereals/grains | Bread, wholegrain or wholemeal | Yeast | 79.31 |
| Bread wholemeal with wheat kernels | Fermented | Cereals/grains | Bread, wholegrain or wholemeal | Yeast | 74.22 |
| Bun wholemeal with muesli | Fermented | Cereals/grains | Bread, wholegrain or wholemeal | Yeast | 67.67 |
| Roll, multigrain, hard | Fermented | Cereals/grains | Bread, wholegrain or wholemeal | Yeast | 72.71 |
| Roll, multigrain, soft | Fermented | Cereals/grains | Bread, wholegrain or wholemeal | Yeast | 56.73 |

| | | | | | |
|---|---|---|---|---|---|
| Roll, w holemeal, soft | Fermented | Cereals/grains | Bread, w holegrain or w holemeal | Yeast | 72.14 |
| Dough for pizza and savoury pie | Fermented | Cereals/grains | Other | Yeast | 91.93 |
| Doughnut, Dutch style | Fermented | Cereals/grains | Other | Yeast | 68.08 |
| Doughnuts, plain | Fermented | Cereals/grains | Other | Yeast | 49.00 |
| Almond filled pastry | Fermented | Cereals/grains | Pastry | Yeast | 47.81 |
| Croissant, average | Fermented | Cereals/grains | Pastry | Yeast | 41.69 |
| Croissant, chocolate | Fermented | Cereals/grains | Pastry | Yeast | 50.33 |
| Croissant prepared w ith butter | Fermented | Cereals/grains | Pastry | Yeast | 45.50 |
| Croissant prepared w ithout butter | Fermented | Cereals/grains | Pastry | Yeast | 45.00 |
| Croissant w ith ham and cheese | Fermented | Cereals/grains | Pastry | Yeast | 65.25 |
| Croissant w ith cheese | Fermented | Cereals/grains | Pastry | Yeast | 44.29 |
| Croissants | Fermented | Cereals/grains | Pastry | Yeast | 47.61 |
| Danish pastry | Fermented | Cereals/grains | Pastry | Yeast | 62.95 |
| Waffle (Luikse) | Fermented | Cereals/grains | Pastry | Yeast | 55.00 |
| Waffle, soft-/sugar-/flash- | Fermented | Cereals/grains | Pastry | Yeast | 43.25 |
| Chocolate bar milk w ith nuts | Fermented | Cocoa-based products | Chocolate | Endogenous microorganisms | 28.32 |
| Chocolate bar milk w ithout sugar | Fermented | Cocoa-based products | Chocolate | Endogenous microorganisms | 6.20 |
| Chocolate, confetti, average | Fermented | Cocoa-based products | Chocolate | Endogenous microorganisms | 14.10 |
| Chocolate, confetti, milk | Fermented | Cocoa-based products | Chocolate | Endogenous microorganisms | 15.01 |
| Chocolate, confetti, mix of w hite and plain | Fermented | Cocoa-based products | Chocolate | Endogenous microorganisms | 14.25 |
| Chocolate, confetti, plain | Fermented | Cocoa-based products | Chocolate | Endogenous microorganisms | 15.52 |
| Chocolate, confetti, w hite | Fermented | Cocoa-based products | Chocolate | Endogenous microorganisms | 11.33 |
| Chocolate flakes, average | Fermented | Cocoa-based products | Chocolate | Endogenous microorganisms | 14.33 |

| | | | | | |
|---|---|---|---|---|---|
| Chocolate flakes, milk | Fermented | Cocoa-based products | Chocolate | Endogenous microorganisms | 13.69 |
| Chocolate flakes, plain | Fermented | Cocoa-based products | Chocolate | Endogenous microorganisms | 16.23 |
| Chocolate liqueurs | Fermented | Cocoa-based products | Chocolate | Endogenous microorganisms | 15.15 |
| Chocolate, plain | Fermented | Cocoa-based products | Chocolate | Endogenous microorganisms | 15.24 |
| Chocolate, plain with nuts | Fermented | Cocoa-based products | Chocolate | Endogenous microorganisms | 20.00 |
| Chocolate, plain without sugar | Fermented | Cocoa-based products | Chocolate | Endogenous microorganisms | 10.67 |
| Chocolate white | Fermented | Cocoa-based products | Chocolate | Endogenous microorganisms | 17.96 |
| Chocolates, filled/Belguim chocolate | Fermented | Cocoa-based products | Chocolate | Endogenous microorganisms | 21.72 |
| Milk chocolate | Fermented | Cocoa-based products | Chocolate | Endogenous microorganisms | 18.33 |
| Milk chocolate with hazelnuts w o sugar | Fermented | Cocoa-based products | Chocolate | Endogenous microorganisms | 48.83 |
| Milk chocolate with puffed rice | Fermented | Cocoa-based products | Chocolate | Endogenous microorganisms | 16.50 |
| Cocoa powder | Fermented | Cocoa-based products | Chocolate, powder | Endogenous microorganisms | 2.33 |
| Buttermilk | Fermented | Dairy | Buttermilk | Bacteria | 211.36 |
| Buttermilk with fruit | Fermented | Dairy | Buttermilk | Bacteria | 207.86 |
| Cheese 10+ | Fermented | Dairy | Cheese, hard | Bacteria | 36.00 |
| Cheese 20+ | Fermented | Dairy | Cheese, hard | Bacteria | 20.96 |
| Cheese 20+, Leiden with cumin/Frisian clove | Fermented | Dairy | Cheese, hard | Bacteria | 32.00 |
| Cheese 20+, rindless | Fermented | Dairy | Cheese, hard | Bacteria | 20.00 |
| Cheese 30+ | Fermented | Dairy | Cheese, hard | Bacteria | 24.45 |
| Cheese 30+, low salt | Fermented | Dairy | Cheese, hard | Bacteria | 21.53 |

| | | | | | |
|---|---|---|---|---|---|
| Cheese 40+, Leiden with cumin/Frisian clove | Fermented | Dairy | Cheese, hard | Bacteria | 27.63 |
| Cheese 40+, sodium reduced | Fermented | Dairy | Cheese, hard | Bacteria | 20.67 |
| Cheese 45+ | Fermented | Dairy | Cheese, hard | Bacteria | 27.68 |
| Cheese 48+, low salt | Fermented | Dairy | Cheese, hard | Bacteria | 22.80 |
| Cheese 50+ | Fermented | Dairy | Cheese, hard | Bacteria | 24.36 |
| Cheese, Amsterdam 48+ | Fermented | Dairy | Cheese, hard | Bacteria | 22.94 |
| Cheese, Bluefort | Fermented | Dairy | Cheese, hard | Bacteria | 33.14 |
| Cheese, Cheddar | Fermented | Dairy | Cheese, hard | Bacteria | 23.50 |
| Cheese, Edam 40+ | Fermented | Dairy | Cheese, hard | Bacteria | 26.31 |
| Cheese, Emmenthaler | Fermented | Dairy | Cheese, hard | Bacteria | 29.02 |
| Cheese, goat, hard | Fermented | Dairy | Cheese, hard | Bacteria | 25.42 |
| Cheese, Gouda 48+, aged 10-12 mths | Fermented | Dairy | Cheese, hard | Bacteria | 27.63 |
| Cheese, Gouda 48+, aged 4-7 mths | Fermented | Dairy | Cheese, hard | Bacteria | 24.71 |
| Cheese, Gouda 48+, aged 4-8 weeks | Fermented | Dairy | Cheese, hard | Bacteria | 25.98 |
| Cheese, Gouda 48+, aged 8 wk-4 mths | Fermented | Dairy | Cheese, hard | Bacteria | 25.16 |
| Cheese, Gouda 48+, average | Fermented | Dairy | Cheese, hard | Bacteria | 25.49 |
| Cheese, Gruyere | Fermented | Dairy | Cheese, hard | Bacteria | 31.60 |
| Cheese, Parmesan | Fermented | Dairy | Cheese, hard | Bacteria | 19.17 |
| Cheese, processed, rindless 40+ | Fermented | Dairy | Cheese, hard | Bacteria | 25.06 |
| Cheese product with vegetable fat 45+ | Fermented | Dairy | Cheese, hard | Bacteria | 17.00 |
| Cheese, raw milk 48+ | Fermented | Dairy | Cheese, hard | Bacteria | 28.17 |
| Cheese, smoked | Fermented | Dairy | Cheese, hard | Bacteria | 20.57 |
| Cheese, Swiss, dried | Fermented | Dairy | Cheese, hard | Bacteria | 6.29 |
| Cheese, Gorgonzola | Fermented | Dairy | Cheese, medium | Bacteria/mold | 43.16 |
| Cheese, Leerdammer/Maasdammer 45+ | Fermented | Dairy | Cheese, medium | Bacteria | 32.87 |

| | | | | | |
|---|---|---|---|---|---|
| Cheese, Roquefort | Fermented | Dairy | Cheese, medium | Bacteria/mold | 27.63 |
| Cheese, Saint Paulin/Port Salut | Fermented | Dairy | Cheese, medium | Bacteria | 40.80 |
| Cheese, Stilton | Fermented | Dairy | Cheese, medium | Bacteria/mold | 28.67 |
| Cheese, Brie 50+ | Fermented | Dairy | Cheese, soft | Bacteria/mold | 22.00 |
| Cheese, Brie 60+ | Fermented | Dairy | Cheese, soft | Bacteria/mold | 30.58 |
| Cheese, Camembert 30+ | Fermented | Dairy | Cheese, soft | Bacteria/mold | 12.94 |
| Cheese, Camembert 45+ | Fermented | Dairy | Cheese, soft | Bacteria/mold | 53.16 |
| Cheese, goat fresh | Fermented | Dairy | Cheese, soft | Bacteria | 33.67 |
| Cheese, Kernhem 60+ | Fermented | Dairy | Cheese, soft | Bacteria | 38.50 |
| Cheese, Rambol | Fermented | Dairy | Cheese, soft | Bacteria | 21.33 |
| Cheese, sheep fresh | Fermented | Dairy | Cheese, soft | Bacteria | 36.07 |
| Cream, sour | Fermented | Dairy | Cream | Bacteria | 24.50 |
| Creme fraiche | Fermented | Dairy | Cream | Bacteria | 24.34 |
| Creme fraiche, half fat | Fermented | Dairy | Cream | Bacteria | 51.33 |
| Fromage frais, full fat | Fermented | Dairy | Quark | Bacteria | 109.81 |
| Fromage frais, full fat with fruit | Fermented | Dairy | Quark | Bacteria | 129.00 |
| Fromage frais, half fat | Fermented | Dairy | Quark | Bacteria | 99.92 |
| Fromage frais, half fat with fruit | Fermented | Dairy | Quark | Bacteria | 121.30 |
| Fromage frais, low fat | Fermented | Dairy | Quark | Bacteria | 122.80 |
| Fromage frais, low fat with fruit with sweetener | Fermented | Dairy | Quark | Bacteria | 180.96 |
| Fromage frais, low fat with fruit | Fermented | Dairy | Quark | Bacteria | 130.00 |
| Fromage frais, with fruit Danoontje | Fermented | Dairy | Quark | Bacteria | 85.71 |
| Fromage frais, yoghurt with fruit | Fermented | Dairy | Quark | Bacteria | 144.84 |
| Whey drink, light Rivella | Fermented | Dairy | Whey drink | NA | 194.55 |
| Whey drink, Taksi with sugar | Fermented | Dairy | Whey drink | NA | 185.42 |
| Whey drink, Taksi with sugar and sweetener | Fermented | Dairy | Whey drink | NA | 223.33 |
| Whey drink, Taksi with sweetener | Fermented | Dairy | Whey drink | NA | 175.00 |
| Yakult | Fermented | Dairy | Yakult | Bacteria | 67.27 |

| | | | | | |
|---|---|---|---|---|---|
| Yakult light | Fermented | Dairy | Yakult | Bacteria | 65.00 |
| Yoghurt, 0% fat with fruit (Activia) | Fermented | Dairy | Yoghurt | Bacteria | 135.58 |
| Yoghurt, creamy- with fruit | Fermented | Dairy | Yoghurt | Bacteria | 118.00 |
| Yoghurt, full fat | Fermented | Dairy | Yoghurt | Bacteria | 137.26 |
| Yoghurt, full fat, natural (Activia) | Fermented | Dairy | Yoghurt | Bacteria | 143.75 |
| Yoghurt, full fat with fruit/muesli (Activia) | Fermented | Dairy | Yoghurt | Bacteria | 125.14 |
| Yoghurt, full fat with fruit | Fermented | Dairy | Yoghurt | Bacteria | 143.14 |
| Yoghurt, Greek, full fat | Fermented | Dairy | Yoghurt | Bacteria | 113.26 |
| Yoghurt, half fat | Fermented | Dairy | Yoghurt | Bacteria | 161.36 |
| Yoghurt, half fat (Vifit naturel) | Fermented | Dairy | Yoghurt | Bacteria | 183.33 |
| Yoghurt, half fat with fruit | Fermented | Dairy | Yoghurt | Bacteria | 138.41 |
| Yoghurt, low fat | Fermented | Dairy | Yoghurt | Bacteria | 154.21 |
| Yoghurt, low fat (Bulgarian) | Fermented | Dairy | Yoghurt | Bacteria | 7.62 |
| Yoghurt, low fat with fruit with sw Vitalinea | Fermented | Dairy | Yoghurt | Bacteria | 164.29 |
| Yoghurt, low fat with fruit/van with sweetner (Optimel) | Fermented | Dairy | Yoghurt | Bacteria | 174.19 |
| Yoghurt, low fat with fruit | Fermented | Dairy | Yoghurt | Bacteria | 142.54 |
| Yoghurt, low fat with fruit with sweeteners | Fermented | Dairy | Yoghurt | Bacteria | 139.67 |
| Yoghurt, vanilla, half fat | Fermented | Dairy | Yoghurt | Bacteria | 144.37 |
| Yoghurt, whole milk (Bulgarian) | Fermented | Dairy | Yoghurt | Bacteria | 66.54 |
| Actimel | Fermented | Dairy | Yoghurt, drink product | Bacteria | 99.22 |
| Actimel light | Fermented | Dairy | Yoghurt, drink product | Bacteria | 112.50 |
| Breakfast drink (Breaker Breakfast) | Fermented | Dairy | Yoghurt, drink product | Bacteria | 200.00 |
| Yoghurt drink | Fermented | Dairy | Yoghurt, drink product | Bacteria | 177.42 |

| | | | | | |
|---|---|---|---|---|---|
| Yoghurt drink (Becel pro-activ) | Fermented | Dairy | Yoghurt, drink product | Bacteria | 112.17 |
| Yoghurt drink (Fristi) | Fermented | Dairy | Yoghurt, drink product | Bacteria | 198.75 |
| Yoghurt drink (Fristi) with sweetners | Fermented | Dairy | Yoghurt, drink product | Bacteria | 130.00 |
| Yoghurt drink (Vifit), fruit | Fermented | Dairy | Yoghurt, drink product | Bacteria | 230.60 |
| Yoghurt drink (Vifit), fruit, light | Fermented | Dairy | Yoghurt, drink product | Bacteria | 249.29 |
| Yoghurt drink (Vifit), natural | Fermented | Dairy | Yoghurt, drink product | Bacteria | 175.00 |
| Yoghurt drink with sweeteners (Optimel) | Fermented | Dairy | Yoghurt, drink product | Bacteria | 207.83 |
| Yoghurt drink with sweetners | Fermented | Dairy | Yoghurt, drink product | Bacteria | 181.25 |
| Yoghurt drink (Yomild), fruit | Fermented | Dairy | Yoghurt, drink product | Bacteria | 222.50 |
| Yoghurt drink (Yomild), naturel | Fermented | Dairy | Yoghurt, drink product | Bacteria | 156.40 |
| Yoghurt product (Becel pro-activ) | Fermented | Dairy | Yoghurt, drink product | Bacteria | 166.67 |
| Yoghurt snack (Breaker) | Fermented | Dairy | Yoghurt, drink product | Bacteria | 200.00 |
| Vegetables, mixed pickled (Atjar tjampoer) | Fermented | Fruits/Vegetables | Other | Bacteria | 68.42 |
| Cabbage, sauerkraut, cooked | Fermented | Fruits/Vegetables | Sauerkraut | Bacteria | 141.94 |
| Cabbage, sauerkraut, raw | Fermented | Fruits/Vegetables | Sauerkraut | Bacteria | 60.91 |
| Juice, sauerkraut | Fermented | Fruits/Vegetables | Sauerkraut | Bacteria | 220.00 |
| Herring, salted | Fermented | Meat/Fish | Fish | Endogenous microorganisms | 101.66 |
| Salami | Fermented | Meat/Fish | Sausages | Bacteria | 24.82 |

| | | | | | |
|---|---|---|---|---|---|
| Salami sausage, saveloy | Fermented | Meat/Fish | Sausages | Bacteria | 23.81 |
| Salami sausage, saveloy, lean | Fermented | Meat/Fish | Sausages | Bacteria | 32.60 |
| Sausage, Chorizo | Fermented | Meat/Fish | Sausages | Bacteria | 32.31 |
| Sausage, dry salami, Turkish | Fermented | Meat/Fish | Sausages | Bacteria | 33.75 |
| Yeast extract, Marmite | Fermented | Other | Marmite | Yeast | 3.56 |
| Vinegar | Fermented | Other | Vinegar | Bacteria/yeast | 8.30 |
| Tempeh, fermented soya beans | Fermented | Soya | Soya | Mold/Bacteria | 49.60 |
| Soya sauce, savoury | Fermented | Soya | Soya sauce | Mold/Bacteria/Yeast | 14.05 |
| Soya sauce, sw eet | Fermented | Soya | Soya sauce | Mold/Bacteria/Yeast | 13.98 |
| Soya sauce, sw eet, low sodium | Fermented | Soya | Soya sauce | Mold/Bacteria/Yeast | 7.00 |
| Kale curly w ith mashed potatoes and sausage | Composite dish/contains a fermented ingredient but is not itself fermented. | NA | NA | NA | 385.00 |
| Fried rice, Nasi goring, frozen/tinned | Composite dish/contains a fermented ingredient but is not itself fermented. | NA | NA | NA | 350.00 |
| Sauerkraut w ith mashed potatoes w ithout meat | Composite dish/contains a fermented ingredient but is not itself fermented. | NA | NA | NA | 342.44 |
| Lasagna w ith meat and sauce, frozen | Composite dish/contains a fermented ingredient but is not itself fermented. | NA | NA | NA | 333.33 |
| Lasagna w ith meat and sauce, ready-to-eat | Composite dish/contains a fermented ingredient but is not itself fermented. | NA | NA | NA | 292.27 |
| Spaghetti Bolognese, frozen | Composite dish/contains a fermented ingredient but is not itself fermented. | NA | NA | NA | 259.00 |
| Porridge, buttermilk w ith w heat flour | Composite dish/contains a fermented ingredient but is not itself fermented. | NA | NA | NA | 255.00 |
| Chinese noodle dish, Bami goreng frozen | Composite dish/contains a fermented ingredient but is not itself fermented. | NA | NA | NA | 253.33 |
| Fried rice, Nasi goreng, ready-to-eat | Composite dish/contains a fermented ingredient but is not itself fermented. | NA | NA | NA | 235.38 |
| Pizza, salami, frozen, unprepared | Composite dish/contains a fermented ingredient but is not itself fermented. | NA | NA | NA | 227.19 |
| Pizza w ith ham and pineapple, frozen, unprepared | Composite dish/contains a fermented ingredient but is not itself fermented. | NA | NA | NA | 223.79 |

| | | | | | |
|---|---|---|---|---|---|
| Chinese noodles, Bami goreng, ready-to-eat | Composite dish/contains a fermented ingredient but is not itself fermented. | NA | NA | NA | 223.06 |
| Pizza with meat frozen, unprepared | Composite dish/contains a fermented ingredient but is not itself fermented. | NA | NA | NA | 220.52 |
| Quiche with vegetables/cheese/egg | Composite dish/contains a fermented ingredient but is not itself fermented. | NA | NA | NA | 219.19 |
| Pizza with cheese and vegetables, frozen | Composite dish/contains a fermented ingredient but is not itself fermented. | NA | NA | NA | 216.86 |
| Smoothie, fruit with dairy | Composite dish/contains a fermented ingredient but is not itself fermented. | NA | NA | NA | 213.33 |
| Pizza with fish, frozen, unprepared | Composite dish/contains a fermented ingredient but is not itself fermented. | NA | NA | NA | 209.50 |
| Fried rice Nasi goreng with egg | Composite dish/contains a fermented ingredient but is not itself fermented. | NA | NA | NA | 203.09 |
| Spinach, tinned | Processed/tinned but unclear if fermented as well. | NA | NA | NA | 200.00 |
| Pizza with frozen meat | Composite dish/contains a fermented ingredient but is not itself fermented. | NA | NA | NA | 195.17 |
| Chinese noodle, dish Bami goreng, without egg | Composite dish/contains a fermented ingredient but is not itself fermented. | NA | NA | NA | 186.59 |
| Pizza with cheese and tomato | Composite dish/contains a fermented ingredient but is not itself fermented. | NA | NA | NA | 181.23 |
| Pizza with frozen vegetables | Composite dish/contains a fermented ingredient but is not itself fermented. | NA | NA | NA | 175.66 |
| Beans, baked in tomato sauce, tinned | Processed/tinned but unclear if fermented as well. | NA | NA | NA | 170.83 |
| Spinach, creamed, frozen, boiled | Composite dish/contains a fermented ingredient but is not itself fermented. | NA | NA | NA | 167.15 |
| Hot chocolate from vending machine | Composite dish/contains a fermented ingredient but is not itself fermented. | NA | NA | NA | 165.37 |
| Peas, marrow fat legumes, tinned | Processed/tinned but unclear if fermented as well. | NA | NA | NA | 164.82 |
| Pancake | Composite dish/contains a fermented ingredient but is not itself fermented. | NA | NA | NA | 161.93 |

| | | | | | |
|---|---|---|---|---|---|
| Tortellini, boiled | Composite dish/contains a fermented ingredient but is not itself fermented. | NA | NA | NA | 158.00 |
| Peas and carrots, tinned | Processed/tinned but unclear if fermented as well. | NA | NA | NA | 156.53 |
| Peas, garden extra fine, low sodium, tinned | Processed/tinned but unclear if fermented as well. | NA | NA | NA | 150.00 |
| Porridge, buttermilk groats | Composite dish/contains a fermented ingredient but is not itself fermented. | NA | NA | NA | 148.33 |
| Tart with bread base with veg/egg/cheese | Composite dish/contains a fermented ingredient but is not itself fermented. | NA | NA | NA | 141.08 |
| Carrots, tinned | Processed/tinned but unclear if fermented as well. | NA | NA | NA | 135.92 |
| Ragout with meat, all types | Composite dish/contains a fermented ingredient but is not itself fermented. | NA | NA | NA | 132.00 |
| Beans, brown, tinned | Processed/tinned but unclear if fermented as well. | NA | NA | NA | 130.72 |
| Pizza with cross-bottom, frozen | Composite dish/contains a fermented ingredient but is not itself fermented. | NA | NA | NA | 125.00 |
| Mackerel in oil, tinned | Processed/tinned but unclear if fermented as well. | NA | NA | NA | 125.00 |
| Beetroot, pickled, glass | Pickled (probably with vinegar) but unclear if fermented. | NA | NA | NA | 123.75 |
| Dessert made of custard, yoghurt & syrup | Composite dish/contains a fermented ingredient but is not itself fermented. | NA | NA | NA | 121.78 |
| Tomatoes, tinned | Processed/tinned but unclear if fermented as well. | NA | NA | NA | 121.52 |
| Pork, Chinese style, Babi pangang, without rice | Composite dish/contains a fermented ingredient but is not itself fermented. | NA | NA | NA | 121.03 |
| Cabbage, red, glass | Processed/tinned but unclear if fermented as well. | NA | NA | NA | 120.55 |
| Macaroni with ham & cheese sauce, tinned | Composite dish/contains a fermented ingredient but is not itself fermented. | NA | NA | NA | 120.00 |
| Strawberry puree, tinned | Processed/tinned but unclear if fermented as well. | NA | NA | NA | 120.00 |

| | | | | | |
|---|---|---|---|---|---|
| Beans, French, tinned | Processed/tinned but unclear if fermented as well. | NA | NA | NA | 117.06 |
| Peas, garden, medium fine, tinned | Processed/tinned but unclear if fermented as well. | NA | NA | NA | 116.84 |
| Croquettes, potato, frozen, unprepared | Composite dish/contains a fermented ingredient but is not itself fermented. | NA | NA | NA | 115.75 |
| Pears in syrup, tinned | Processed/tinned but unclear if fermented as well. | NA | NA | NA | 113.39 |
| Herring fillet in tomato sauce, tinned | Processed/tinned but unclear if fermented as well. | NA | NA | NA | 110.67 |
| Herring, smoked | Processed/tinned but unclear if fermented as well. | NA | NA | NA | 110.00 |
| Cheesecake made with fromage frais | Composite dish/contains a fermented ingredient but is not itself fermented. | NA | NA | NA | 108.66 |
| Pea, garden super fine, tinned | Processed/tinned but unclear if fermented as well. | NA | NA | NA | 108.00 |
| Beans, broad, tinned | Processed/tinned but unclear if fermented as well. | NA | NA | NA | 104.00 |
| Raisins coated with milk chocolate | Composite dish/contains a fermented ingredient but is not itself fermented. | NA | NA | NA | 100.67 |
| Apple sauce, tinned | Processed/tinned but unclear if fermented as well. | NA | NA | NA | 99.93 |
| Chocolate pudding with sauce | Composite dish/contains a fermented ingredient but is not itself fermented. | NA | NA | NA | 98.33 |
| Salad, Russian | Composite dish/contains a fermented ingredient but is not itself fermented. | NA | NA | NA | 89.99 |
| Chop-suey, Indonesian, without rice | Composite dish/contains a fermented ingredient but is not itself fermented. | NA | NA | NA | 89.93 |
| Tangerines in syrup, tinned | Processed/tinned but unclear if fermented as well. | NA | NA | NA | 89.54 |
| Herring, pickled with gherkin | Composite dish/contains a fermented ingredient but is not itself fermented. | NA | NA | NA | 87.33 |
| Snack sausage roll with bread, dough pastry | Composite dish/contains a fermented ingredient but is not itself fermented. | NA | NA | NA | 84.00 |

| | | | | | |
|---|---|---|---|---|---|
| Peaches in syrup, tinned | Processed/tinned but unclear if fermented as well. | NA | NA | NA | 83.72 |
| Apricots in syrup, tinned | Processed/tinned but unclear if fermented as well. | NA | NA | NA | 80.00 |
| Plums in syrup, tinned | Processed/tinned but unclear if fermented as well. | NA | NA | NA | 80.00 |
| Apple sauce without sugar, tinned | Processed/tinned but unclear if fermented as well. | NA | NA | NA | 78.16 |
| Herring, pickled (sweet and sour) | Composite dish/contains a fermented ingredient but is not itself fermented. | NA | NA | NA | 77.85 |
| Fruit cocktail in syrup, tinned | Processed/tinned but unclear if fermented as well. | NA | NA | NA | 76.58 |
| Foe jung hai, filled omelette | Composite dish/contains a fermented ingredient but is not itself fermented. | NA | NA | NA | 73.84 |
| Salmon, tinned | Processed/tinned but unclear if fermented as well. | NA | NA | NA | 72.69 |
| Mussels, pickled | Composite dish/contains a fermented ingredient but is not itself fermented. | NA | NA | NA | 72.33 |
| Gammon, boiled, deboned | Processed/tinned but unclear if fermented as well. | NA | NA | NA | 71.81 |
| Sauce, warm liquid, ready-made (<12% fat) | Composite dish/contains a fermented ingredient but is not itself fermented. | NA | NA | NA | 71.62 |
| Chocolate eclair | Composite dish/contains a fermented ingredient but is not itself fermented. | NA | NA | NA | 70.00 |
| Mushrooms, tinned | Processed/tinned but unclear if fermented as well. | NA | NA | NA | 69.75 |
| Cucumber, sliced, pickled | Pickled (probably with vinegar) but unclear if fermented. | NA | NA | NA | 68.50 |
| Tuna in oil, tinned | Processed/tinned but unclear if fermented as well. | NA | NA | NA | 68.15 |
| Sardines/pilchards in oil, tinned | Processed/tinned but unclear if fermented as well. | NA | NA | NA | 66.75 |
| Mackerel in water, tinned | Processed/tinned but unclear if fermented as well. | NA | NA | NA | 65.96 |

| | | | | | |
|---|---|---|---|---|---|
| Flan filling, tinned | Processed/tinned but unclear if fermented as well. | NA | NA | NA | 65.00 |
| Asparagus, tinned | Processed/tinned but unclear if fermented as well. | NA | NA | NA | 63.50 |
| Teacakes, chocolate-coated marshmellow | Composite dish/contains a fermented ingredient but is not itself fermented. | NA | NA | NA | 63.00 |
| Roti Surinam pancake | Composite dish/contains a fermented ingredient but is not itself fermented. | NA | NA | NA | 62.00 |
| Tuna in water, tinned | Processed/tinned but unclear if fermented as well. | NA | NA | NA | 61.82 |
| Cherries in syrup, tinned | Processed/tinned but unclear if fermented as well. | NA | NA | NA | 59.56 |
| Snack sausage roll with bread dough frozen | Composite dish/contains a fermented ingredient but is not itself fermented. | NA | NA | NA | 56.50 |
| Sauce, cheese | Processed cheese. | NA | NA | NA | 56.04 |
| Biscuits, fortified (Fruitkick Extra) | Unclear if yeast or another leavening agent is used. | NA | NA | NA | 54.00 |
| Advocaat liqueur | Composite dish/contains a fermented ingredient but is not itself fermented. | NA | NA | NA | 53.41 |
| Biscuits, spiced Speculaas with almond paste | Unclear if yeast or another leavening agent is used. | NA | NA | NA | 51.46 |
| Sauce, warm liquid, ready-made (>12% fat) | Composite dish/contains a fermented ingredient but is not itself fermented. | NA | NA | NA | 50.50 |
| Processed meat product, low sodium, average | Unclear if processing is fermentation or another method. | NA | NA | NA | 50.00 |
| Sauce, oriental, ready-made in jar/bag | Composite dish/contains a fermented ingredient but is not itself fermented. | NA | NA | NA | 47.67 |
| Pineapple in syrup, tinned | Processed/tinned but unclear if fermented as well. | NA | NA | NA | 47.58 |
| Cake wrapped in marzipan and chocolate | Composite dish/contains a fermented ingredient but is not itself fermented. | NA | NA | NA | 46.23 |
| Luncheon meat, tinned | Processed/tinned but unclear if fermented as well. | NA | NA | NA | 46.00 |
| Muesli, crunchy with chocolate | Composite dish/contains a fermented ingredient but is not itself fermented. | NA | NA | NA | 44.80 |

| | | | | | |
|---|---|---|---|---|---|
| Eel, smoked | Processed/tinned but unclear if fermented as well. | NA | NA | NA | 44.00 |
| Sweet pepper, sweet/sour, pickled | Pickled (probably with vinegar) but unclear if fermented. | NA | NA | NA | 43.55 |
| Pastry puff cheese, filled, unprepared | Composite dish/contains a fermented ingredient but is not itself fermented. | NA | NA | NA | 43.54 |
| Mango chutney | Composite dish/contains a fermented ingredient but is not itself fermented. | NA | NA | NA | 42.00 |
| Biscuits, oatmeal | Unclear if yeast or another leavening agent is used. | NA | NA | NA | 41.17 |
| Fruit in syrup, tinned | Processed/tinned but unclear if fermented as well. | NA | NA | NA | 40.07 |
| Biscuits fortified with currants (LigaEvergreen) | Unclear if yeast or another leavening agent is used. | NA | NA | NA | 38.63 |
| Sauce Chinese, sweet & sour, home-made preparation | Composite dish/contains a fermented ingredient but is not itself fermented. | NA | NA | NA | 38.34 |
| Biscuits, Jaffa cakes/Cake (PiM's) | Unclear if yeast or another leavening agent is used. | NA | NA | NA | 38.00 |
| Cake, chocolate made with butter | Composite dish/contains a fermented ingredient but is not itself fermented. | NA | NA | NA | 37.88 |
| Biscuits, fortified (Liga Milkbreak) | Unclear if yeast or another leavening agent is used. | NA | NA | NA | 37.50 |
| M&M's, chocolate with peanuts | Composite dish/contains a fermented ingredient but is not itself fermented. | NA | NA | NA | 37.43 |
| Raspberry/red currant puree, tinned | Processed/tinned but unclear if fermented as well. | NA | NA | NA | 36.67 |
| Sausage excluding liver, average | Unclear if fermented. | NA | NA | NA | 34.89 |
| Bamboo shoots, tinned | Processed/tinned but unclear if fermented as well. | NA | NA | NA | 34.63 |
| Biscuits, fruit | Unclear if yeast or another leavening agent is used. | NA | NA | NA | 33.67 |
| Candybar, KitKat | Composite dish/contains a fermented ingredient but is not itself fermented. | NA | NA | NA | 33.35 |
| Biscuits, fortified (Liga Evergreen? assortment) | Unclear if yeast or another leavening agent is used. | NA | NA | NA | 31.92 |

| | | | | | |
|---|---|---|---|---|---|
| Tomatoes dried in oil tin/glass | Processed/tinned but unclear if fermented as well. | NA | NA | NA | 31.90 |
| Candybar, Twix | Composite dish/contains a fermented ingredient but is not itself fermented. | NA | NA | NA | 31.73 |
| Sausage raw beef (ossenworst) | Unclear if fermented. | NA | NA | NA | 31.33 |
| Candybar, Snickers | Composite dish/contains a fermented ingredient but is not itself fermented. | NA | NA | NA | 30.62 |
| Gherkins, sweet, pickled | Pickled (probably with vinegar) but unclear if fermented. | NA | NA | NA | 30.28 |
| Candybar, Bounty | Composite dish/contains a fermented ingredient but is not itself fermented. | NA | NA | NA | 29.67 |
| Cake made with butter | Composite dish/contains a fermented ingredient but is not itself fermented. | NA | NA | NA | 29.63 |
| Cake, butter, Dutch (Boterkoek) | Composite dish/contains a fermented ingredient but is not itself fermented. | NA | NA | NA | 29.42 |
| Sauce, barbecue | Composite dish/contains a fermented ingredient but is not itself fermented. | NA | NA | NA | 29.41 |
| Eclair, cheese cream filled | Composite dish/contains a fermented ingredient but is not itself fermented. | NA | NA | NA | 29.33 |
| Sauce (Joppie) | Composite dish/contains a fermented ingredient but is not itself fermented. | NA | NA | NA | 28.75 |
| Herb paste (boemboe) | Composite dish/contains a fermented ingredient but is not itself fermented. | NA | NA | NA | 27.74 |
| Biscuits, chocolate-coated (Chocoprins) | Unclear if yeast or another leavening agent is used. | NA | NA | NA | 27.16 |
| Beef, salted, cooked | Processed/tinned but unclear if fermented as well. | NA | NA | NA | 27.00 |
| Sauce for chips (35% oil) | Composite dish/contains a fermented ingredient but is not itself fermented. | NA | NA | NA | 26.73 |
| Biscuits with dried fruit & yoghurt (Yofruit) | Unclear if yeast or another leavening agent is used. | NA | NA | NA | 25.89 |
| Tomato puree, concentrated, tinned | Processed/tinned but unclear if fermented as well. | NA | NA | NA | 25.85 |
| Biscuits, filled (Prince) | Unclear if yeast or another leavening agent is used. | NA | NA | NA | 25.75 |

| | | | | | |
|---|---|---|---|---|---|
| Biscuits & snacks, cheesy, average | Unclear if yeast or another leavening agent is used. | NA | NA | NA | 25.36 |
| Bacon rashers, streaky | Processed/tinned but unclear if fermented as well. | NA | NA | NA | 25.18 |
| Ketchup, curry | Composite dish/contains a fermented ingredient but is not itself fermented. | NA | NA | NA | 25.16 |
| Onions, silverskin, sweet, pickled without sugar | Pickled (probably with vinegar) but unclear if fermented. | NA | NA | NA | 25.00 |
| Syrup from tinned fruit | Processed/tinned but unclear if fermented as well. | NA | NA | NA | 24.75 |
| Gherkins, sour, pickled | Pickled (probably with vinegar) but unclear if fermented. | NA | NA | NA | 24.69 |
| Cheese spread 60+ (Kiri) | Processed cheese. | NA | NA | NA | 24.50 |
| Ketchup, tomato | Composite dish/contains a fermented ingredient but is not itself fermented. | NA | NA | NA | 24.38 |
| Sauce, cocktail/party/table (>25% oil) | Composite dish/contains a fermented ingredient but is not itself fermented. | NA | NA | NA | 23.93 |
| Biscuits, fortified (Liga Fruitkick) | Unclear if yeast or another leavening agent is used. | NA | NA | NA | 23.50 |
| Olives, tinned/glass | Pickled (probably with vinegar) but unclear if fermented. | NA | NA | NA | 23.44 |
| Salad dressing (20% oil with yogurt) | Composite dish/contains a fermented ingredient but is not itself fermented. | NA | NA | NA | 23.38 |
| Biscuits, salted, average | Unclear if yeast or another leavening agent is used. | NA | NA | NA | 23.15 |
| Cheese spread 45+ | Processed cheese. | NA | NA | NA | 23.00 |
| Dairy spread, plain/herbs light | Processed cheese. | NA | NA | NA | 22.98 |
| Sausage including liver products, average | Unclear if fermented. | NA | NA | NA | 22.80 |
| Muesli bar with chocolate | Composite dish/contains a fermented ingredient but is not itself fermented. | NA | NA | NA | 22.80 |
| Bilberries in syrup tinned | Processed/tinned but unclear if fermented as well. | NA | NA | NA | 22.33 |

| | | | | | |
|---|---|---|---|---|---|
| Salad dressing/sauce, approximately 13% oil | Composite dish/contains a fermented ingredient but is not itself fermented. | NA | NA | NA | 22.26 |
| Sauce, chocolate, for pudding | Composite dish/contains a fermented ingredient but is not itself fermented. | NA | NA | NA | 21.58 |
| Liver paté | Processed/tinned but unclear if fermented as w ell. | NA | NA | NA | 21.55 |
| Cheese spread 40+ | Processed cheese. | NA | NA | NA | 21.50 |
| Biscuits, Dutch, shortbread (spritsstukken) | Unclear if yeast or another leavening agent is used. | NA | NA | NA | 21.37 |
| Salad cream (25% oil) | Composite dish/contains a fermented ingredient but is not itself fermented. | NA | NA | NA | 21.26 |
| Biscuits, muesli | Unclear if yeast or another leavening agent is used. | NA | NA | NA | 21.13 |
| Salad dressing, honey/mustard | Composite dish/contains a fermented ingredient but is not itself fermented. | NA | NA | NA | 20.64 |
| Paté, averag | Processed/tinned but unclear if fermented as w ell. | NA | NA | NA | 20.50 |
| Cheese spread 30+ | Processed cheese. | NA | NA | NA | 20.17 |
| Sauce, cocktail/party/table (25% oil) | Composite dish/contains a fermented ingredient but is not itself fermented. | NA | NA | NA | 19.94 |
| Mayonnaise, low fat (40% oil) | Composite dish/contains a fermented ingredient but is not itself fermented. | NA | NA | NA | 19.86 |
| Biscuits, brow n/w holemeal | Unclear if yeast or another leavening agent is used. | NA | NA | NA | 19.66 |
| Biscuits w ith chocolate layer (Scholiertje) | Unclear if yeast or another leavening agent is used. | NA | NA | NA | 19.50 |
| Capers | Pickled (probably w ith vinegar) but unclear if fermented. | NA | NA | NA | 18.85 |
| Ham, smoked, raw | Unclear if fermented. | NA | NA | NA | 18.81 |
| Sauce for chips (25% oil) | Composite dish/contains a fermented ingredient but is not itself fermented. | NA | NA | NA | 18.77 |
| Cocoa pow der, sw eetened (Nesquik Plus) | Composite dish/contains a fermented ingredient but is not itself fermented. | NA | NA | NA | 18.50 |

| | | | | | |
|---|---|---|---|---|---|
| Biscuits, sugar free | Unclear if yeast or another leavening agent is used. | NA | NA | NA | 18.43 |
| Dairy spread, plain/herbs | Processed cheese. | NA | NA | NA | 18.33 |
| Chocolate chip cookie | Composite dish/contains a fermented ingredient but is not itself fermented. | NA | NA | NA | 18.31 |
| Cheese spread 48+ | Processed cheese. | NA | NA | NA | 18.31 |
| Spread, chocolate hazelnut | Composite dish/contains a fermented ingredient but is not itself fermented. | NA | NA | NA | 18.28 |
| Corned beef | Processed/tinned but unclear if fermented as well. | NA | NA | NA | 18.27 |
| Cheese spread 20+ | Processed cheese. | NA | NA | NA | 18.10 |
| Biscuits, chocolate | Unclear if yeast or another leavening agent is used. | NA | NA | NA | 17.98 |
| Sausage luncheon meat (boterham worst) | Unclear if fermented. | NA | NA | NA | 17.70 |
| M&M's chocolate | Composite dish/contains a fermented ingredient but is not itself fermented. | NA | NA | NA | 17.69 |
| Tapenade olive | Pickled (probably with vinegar) but unclear if fermented. | NA | NA | NA | 17.55 |
| Salad dressing (40% oil) (Becel) | Composite dish/contains a fermented ingredient but is not itself fermented. | NA | NA | NA | 17.40 |
| Mayonnaise, yoghurt-based (25% oil) | Composite dish/contains a fermented ingredient but is not itself fermented. | NA | NA | NA | 17.32 |
| Mayonnaise | Composite dish/contains a fermented ingredient but is not itself fermented. | NA | NA | NA | 17.30 |
| Biscuits, digestive with chocolate | Unclear if yeast or another leavening agent is used. | NA | NA | NA | 17.00 |
| Spread, chocolate, milk | Composite dish/contains a fermented ingredient but is not itself fermented. | NA | NA | NA | 16.94 |
| Processed meat products, 10-20 g fat/100 g | Unclear if processing is fermentation or another method. | NA | NA | NA | 16.93 |
| Processed meat products, average | Processed/tinned but unclear if fermented as well. | NA | NA | NA | 16.67 |

| | | | | | |
|---|---|---|---|---|---|
| Biscuits, Dutch (krakeling) | Unclear if yeast or another leavening agent is used. | NA | NA | NA | 16.52 |
| Biscuits, spiced (Speculaas) | Unclear if yeast or another leavening agent is used. | NA | NA | NA | 16.38 |
| Melon in syrup, canned | Processed/tinned but unclear if fermented as w ell. | NA | NA | NA | 16.00 |
| Spread, chocolate, plain | Composite dish/contains a fermented ingredient but is not itself fermented. | NA | NA | NA | 15.95 |
| Biscuits, shortbread, Bastogne | Unclear if yeast or another leavening agent is used. | NA | NA | NA | 15.88 |
| Salad dressing / vinaigrette | Composite dish/contains a fermented ingredient but is not itself fermented. | NA | NA | NA | 15.82 |
| Biscuits, fortified (Liga Continue vitamin) | Unclear if yeast or another leavening agent is used. | NA | NA | NA | 15.75 |
| Spread, chocolate (Duo Penotti hazelnut) | Composite dish/contains a fermented ingredient but is not itself fermented. | NA | NA | NA | 15.35 |
| Beans, runner, tinned | Processed/tinned but unclear if fermented as w ell. | NA | NA | NA | 15.00 |
| Processed meat products (>30 g fat/ 100g) | Unclear if processing is fermentation or another method. | NA | NA | NA | 15.00 |
| Processed meat products (20-30 g fat/100g) | Unclear if processing is fermentation or another method. | NA | NA | NA | 15.00 |
| Syrup, malt barley | Fermentation ingredient but not fermented itself. | NA | NA | NA | 15.00 |
| Spread, duo w ith chocolate | Composite dish/contains a fermented ingredient but is not itself fermented. | NA | NA | NA | 14.79 |
| Piccalilly (South Asian pickles product) | Composite dish/contains a fermented ingredient but is not itself fermented. | NA | NA | NA | 14.75 |
| Forcemeat balls for soup, canned | Processed/tinned but unclear if fermented as w ell. | NA | NA | NA | 14.50 |
| Braw n, pork pickled in vinegar | Composite dish/contains a fermented ingredient but is not itself fermented. | NA | NA | NA | 14.35 |
| Biscuits, sponge fingers | Unclear if yeast or another leavening agent is used. | NA | NA | NA | 14.33 |
| Cocoa pow der, sw eetened (Benco) | Composite dish/contains a fermented ingredient but is not itself fermented. | NA | NA | NA | 14.25 |

| | | | | | |
|---|---|---|---|---|---|
| Candybar, Milky Way | Composite dish/contains a fermented ingredient but is not itself fermented. | NA | NA | NA | 14.20 |
| Cocoa product, powder (Ovomaltine) | Composite dish/contains a fermented ingredient but is not itself fermented. | NA | NA | NA | 14.00 |
| Maltesers | Composite dish/contains a fermented ingredient but is not itself fermented. | NA | NA | NA | 14.00 |
| Biscuits, assorted with butter | Unclear if yeast or another leavening agent is used. | NA | NA | NA | 13.85 |
| Pork side, cured and smoked (casselerrib) | Unclear if fermented. | NA | NA | NA | 13.83 |
| Salad dressing, naturel, without oil | Composite dish/contains a fermented ingredient but is not itself fermented. | NA | NA | NA | 13.78 |
| Biscuits, average | Unclear if yeast or another leavening agent is used. | NA | NA | NA | 13.74 |
| Anchovy in oil, canned | Processed/tinned but unclear if fermented as well. | NA | NA | NA | 13.57 |
| Toffee with chocolate | Composite dish/contains a fermented ingredient but is not itself fermented. | NA | NA | NA | 13.29 |
| Crab in water, tinned | Processed/tinned but unclear if fermented as well. | NA | NA | NA | 12.94 |
| Chocolate spread, white | Composite dish/contains a fermented ingredient but is not itself fermented. | NA | NA | NA | 12.50 |
| Mayonnaise product (approximately 35% oil) | Composite dish/contains a fermented ingredient but is not itself fermented. | NA | NA | NA | 12.20 |
| Beef, smoke-dried | Unclear if fermented. | NA | NA | NA | 12.18 |
| Silver-skin onions, sweet, pickled, glass | Pickled (probably with vinegar) but unclear if fermented. | NA | NA | NA | 11.92 |
| Biscuits, sweet | Unclear if yeast or another leavening agent is used. | NA | NA | NA | 11.68 |
| After eight chocolate mints | Composite dish/contains a fermented ingredient but is not itself fermented. | NA | NA | NA | 11.25 |
| Biscuits, gluten-free (Glutafin) | Unclear if yeast or another leavening agent is used. | NA | NA | NA | 11.00 |
| Ginger stem in syrup, tinned | Processed/tinned but unclear if fermented as well. | NA | NA | NA | 9.00 |

| | | | | | |
|---|---|---|---|---|---|
| Ketchup, spicy | Composite dish/contains a fermented ingredient but is not itself fermented. | NA | NA | NA | 8.79 |
| Biscuits, Dutch (Frou frou) | Unclear if yeast or another leavening agent is used. | NA | NA | NA | 8.21 |
| Mustard | Composite dish/contains a fermented ingredient but is not itself fermented. | NA | NA | NA | 7.43 |
| Pepper, red hot paste | Composite dish/contains a fermented ingredient but is not itself fermented. | NA | NA | NA | 4.49 |
| Pepper, red hot, fried paste | Composite dish/contains a fermented ingredient but is not itself fermented. | NA | NA | NA | 2.00 |

NA = not applicable

[a] Ordered by fermentation status, food group, then subgroup. The following items are included in this table: foods with a fermentation status of “fermented”, composite dishes containing a fermented ingredient, and foods with unclear fermentation status. Other food items in the FFQ or 24-h recall that were clearly not fermented are not included in this table.

[b] Cheeses could be classified as skim, semi-skim, and full-fat based on the guidelines set by the Dutch Dairy Commodities Act (Overheid.nl. Warenwetbesluit Zuivel). Cheeses with designations of 45+ to 60+ were considered to be full-fat, 10+ to 40+ semi-skim, and ≤10 skim.
