# Supplementary material for: Prevalence of fermented foods in the Dutch adult diet and validation of a food frequency questionnaire for estimating their intake in the NQplus cohort
Source: BMC Nutr. 2020 Dec 3;6:69. doi: 10.1186/s40795-020-00394-z (PMC7712622; doi:10.1186/s40795-020-00394-z)
Supplement: Supplementary file 2 — Additional file 2: Table S2. Mean Individual Percent Difference and Test Statistics from Bland-Altman Analyses for the Validation Sample (n = 809). [file 40795_2020_394_MOESM2_ESM.pdf]

Table S2 Mean Individual Percent Difference and Test Statistics from Bland-Altman Analyses for the Validation Sample (n=809)[a]							
	%Difference in individual means[b]	Bland-Altman Analyses					
		Mean Difference			Regression		
		FFQ - Recall	SD	$P_{difference}$	Intercept	Slope	P_{slope}
Fermented beverages, g/day	17.1	-4.1	250.0	0.644	-12.9	0.01	0.558
Coffee, g/day	19.5	9.3	220.8	0.231	-17.7	0.1	0.035
Beer, g/day	-73.9	-2.9	120.4	0.501	3.7	-0.1	0.004
Wine, g/day	0.3	-10.3	68.1	<0.0001	-0.5	-0.1	<0.0001
Fermented cereals/grains, g/day	0.02	-13.9	45.0	<0.0001	-11.9	-0.01	0.668
Brown bread, g/day	50.9	-0.6	38.3	0.633	-3.6	0.1	0.010
White bread, g/day	-151.2	-9.9	27.4	<0.0001	1.6	-0.8	<0.0001
Wholegrain bread, g/day	-28.0	3.0	50.5	0.090	8.8	-0.1	0.053
Rye bread, g/day	196.8	0.2	11.4	0.693	1.1	-0.3	<0.0001
Other bread, g/day	556.0	0.8	19.8	0.229	2.0	-0.1	0.029
Pastries, g/day	-20.0	-0.4	6.1	0.098	0.8	-0.6	<0.0001
Chocolate, g/day	67.8	-3.4	10.6	<0.0001	-0.2	-0.4	<0.0001
Fermented dairy, g/day	55.0	-4.9	104.0	0.182	2.4	-0.04	0.182
Cheese, g/day	134.8	-0.1	24.7	0.921	-9.3	0.3	<0.0001
Yoghurts, g/day	186.6	11.9	89.7	<0.0001	11.7	0.00	0.941
Quark, g/day	-33.1	-10.9	33.9	<0.0001	-2.2	-1.0	<0.0001
Buttermilk, g/day	15756.6	41.9	74.6	<0.0001	0.9	1.9	<0.0001
Non-fermented dairy, g/day	106.6	15.9	113.3	<0.0001	11.1	0.03	0.311
Butter, g/day	-18.7	0.02	6.1	0.921	-0.9	0.3	<0.0001
Cream, g/day	72.6	-4.9	12.9	<0.0001	-0.2	-0.9	<0.0001
Ice cream, g/day	-7.0	-1.7	14.7	0.001	3.2	-0.7	<0.0001
Milk, g/day	65.3	25.1	112.8	<0.0001	20.2	0.04	0.246
Non-fermented soya, g/day	209.7	-1.8	34.6	0.143	1.9	-0.4	<0.0001

FFQ, food frequency questionnaire; SD, standard deviation.

[a] Bland-Altman analyses were performed using the mean energy-adjusted intake values for each food group.

[b] Percent difference is calculated using [(FFQ - Recall)/Recall] x 100% for each individual and subsequently averaged for the entire group.
