# Supplementary material for: Prevalence of fermented foods in the Dutch adult diet and validation of a food frequency questionnaire for estimating their intake in the NQplus cohort
Source: BMC Nutr. 2020 Dec 3;6:69. doi: 10.1186/s40795-020-00394-z (PMC7712622; doi:10.1186/s40795-020-00394-z)
Supplement: Supplementary file 3 — Additional file 3: Table S3. Attenuation Factors for the Reference 24-h Recall Compared to the Food Frequency Questionnaire. [file 40795_2020_394_MOESM3_ESM.pdf]

Table S3 Attenuation Factors for the Reference 24-hour Recall Compared to the Food Frequency Questionnaire

	Attenuation Factors (λx)[a]													
	Crude							Energy-Adjusted						
	Number of 24-h Recalls							Number of 24-h Recalls						
**Food Group**	**2**	**3**	**4**	**5**	**6**	**7**	**8**	**2**	**3**	**4**	**5**	**6**	**7**	**8**
Fermented beverages	0.83	0.88	0.91	0.93	0.94	0.95	0.96	0.81	0.87	0.9	0.92	0.93	0.94	0.95
Coffee	0.86	0.91	0.93	0.95	0.96	0.96	0.97	0.85	0.90	0.92	0.94	0.95	0.96	0.96
Beer	0.71	0.82	0.89	0.93	0.96	0.99	1.00	0.60	0.69	0.74	0.77	0.80	0.82	0.83
Wine	0.65	0.75	0.82	0.86	0.89	0.92	0.94	0.64	0.74	0.80	0.84	0.87	0.89	0.90
Fermented cereals/grains	0.58	0.67	0.73	0.76	0.79	0.81	0.83	0.51	0.6	0.66	0.71	0.74	0.77	0.79
Brown bread	0.32	0.43	0.52	0.6	0.67	0.72	0.77	0.28	0.37	0.44	0.50	0.54	0.58	0.62
White bread	0.27	0.35	0.42	0.47	0.52	0.56	0.59	0.26	0.33	0.39	0.44	0.48	0.51	0.53
Wholegrain bread	0.47	0.59	0.67	0.73	0.78	0.82	0.85	0.47	0.58	0.66	0.71	0.76	0.79	0.82
Rye bread	0.59	0.71	0.8	0.86	0.91	0.94	0.97	0.54	0.63	0.69	0.74	0.77	0.79	0.81
Other bread	0.19	0.27	0.35	0.41	0.47	0.53	0.57	0.20	0.28	0.35	0.41	0.46	0.51	0.56
Pastries	0.23	0.33	0.42	0.5	0.58	0.65	0.72	0.25	0.34	0.43	0.51	0.57	0.63	0.69
Chocolate	0.32	0.41	0.48	0.54	0.59	0.62	0.65	0.33	0.41	0.48	0.53	0.56	0.60	0.62
Fermented dairy	0.52	0.59	0.64	0.67	0.70	0.71	0.73	0.60	0.70	0.76	0.8	0.83	0.86	0.88
Cheeses	0.42	0.53	0.61	0.67	0.71	0.75	0.78	0.41	0.52	0.59	0.65	0.7	0.73	0.76
Yoghurts	0.53	0.67	0.76	0.83	0.88	0.93	0.96	0.53	0.66	0.75	0.82	0.87	0.92	0.95
Quark	0.15	0.18	0.21	0.22	0.23	0.24	0.25	0.14	0.17	0.20	0.21	0.22	0.23	0.24
Buttermilk[b]	-	-	-	-	-	-	-	-	-	-	-	-	-	-
Non-fermented dairy	0.69	0.79	0.86	0.9	0.93	0.96	0.98	0.69	0.79	0.85	0.89	0.92	0.94	0.96
Butter	0.65	0.77	0.84	0.89	0.92	0.95	0.97	0.64	0.74	0.81	0.86	0.89	0.91	0.94
Cream	0.33	0.39	0.43	0.45	0.47	0.49	0.50	0.32	0.38	0.42	0.45	0.47	0.48	0.49
Ice cream	0.20	0.29	0.37	0.44	0.51	0.57	0.63	0.18	0.26	0.33	0.39	0.45	0.50	0.55
Milk	0.57	0.63	0.66	0.69	0.7	0.71	0.72	0.57	0.63	0.66	0.69	0.70	0.71	0.72
Non-fermented soya	0.6	0.66	0.7	0.72	0.74	0.75	0.76	0.61	0.67	0.70	0.72	0.74	0.75	0.76

[a] Crude and energy-adjusted attenuation factors reported for each number of 24-h recalls, ranging from 2 to 8.
[b] For buttermilk, the error model did not converge due to the low variance of the person-specific biases compared to the within- and between-person variances and are therefore not reported.
