# Supplementary material for: Prevalence of fermented foods in the Dutch adult diet and validation of a food frequency questionnaire for estimating their intake in the NQplus cohort
Source: BMC Nutr. 2020 Dec 3;6:69. doi: 10.1186/s40795-020-00394-z (PMC7712622; doi:10.1186/s40795-020-00394-z)
Supplement: Supplementary file 4 — Additional file 4: Figure S1. Bland-Altman plots demonstrating relative validity of the FFQ versus 24-h recalls for fermented food subgroups. [file 40795_2020_394_MOESM4_ESM.pdf]

Figure S1. Bland-Altman plots demonstrating relative validity of the FFQ versus 24-h recalls for fermented food subgroups. Group-level relative validity shown for the following subgroups: **(a)** beer, **(b)** coffee, **(c)** wine, **(d)** brown bread, **(e)** white bread, **(f)** wholegrain and wholemeal bread, **(g)** rye bread, **(h)** other bread, **(i)** pastries, **(j)** cheeses, **(k)** yoghurts, **(l)** quark, and **(m)** buttermilk. The middle line indicates the mean difference, while the upper and lower lines indicate the 95% confidence intervals, respectively [calculated as: mean ± (standard deviation of the mean difference X 1.96)].

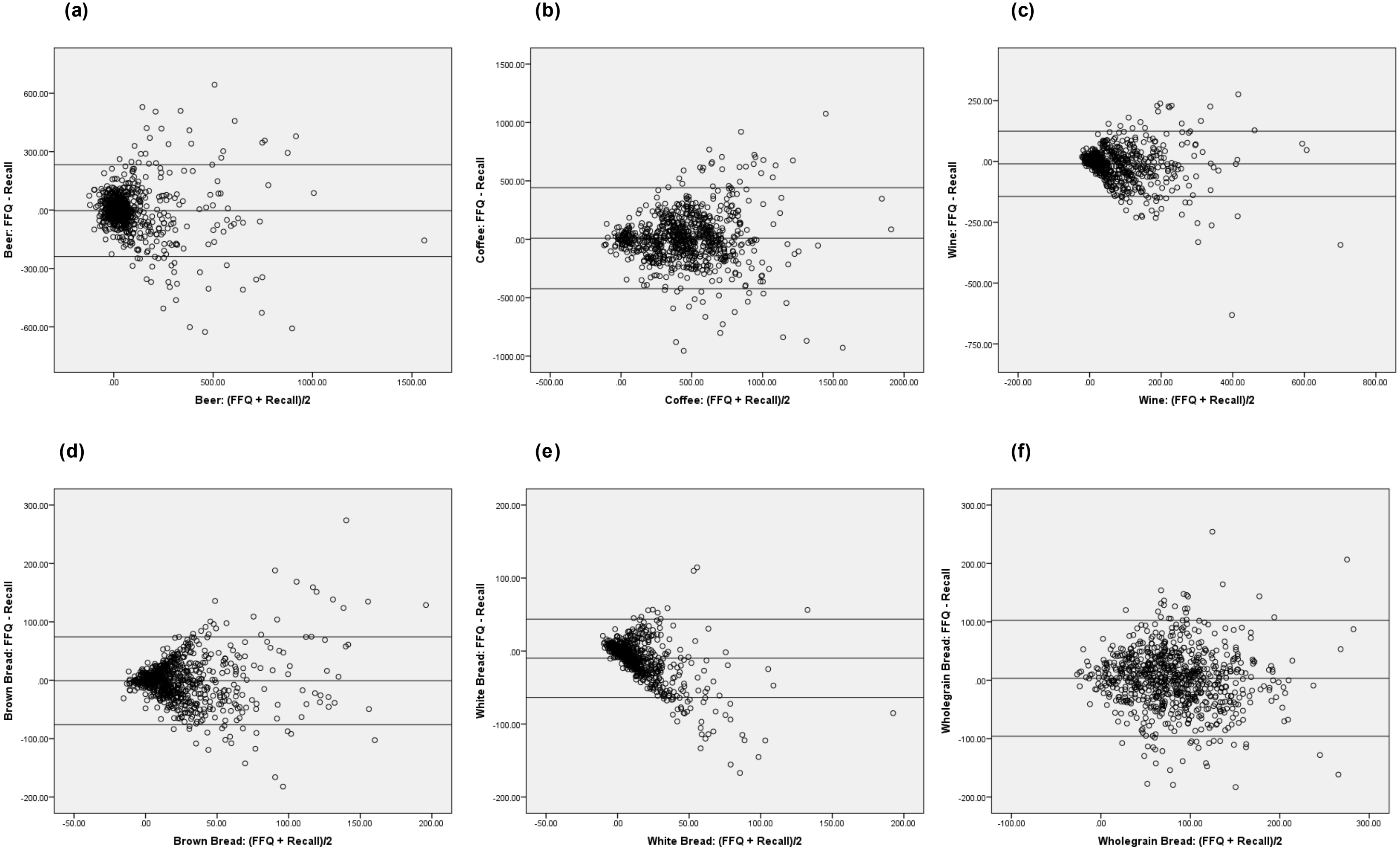

(g)

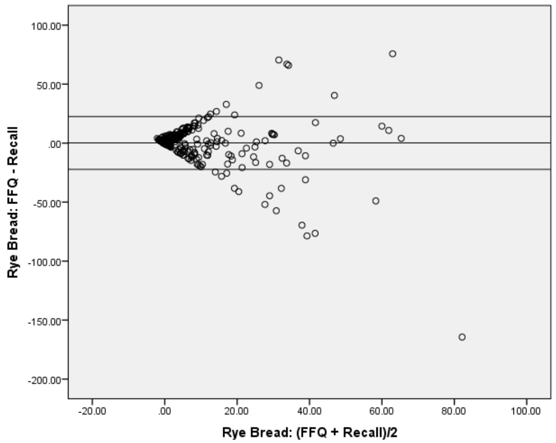

Rye Bread: FFQ - Recall
100.00
50.00
.00
-50.00
-100.00
-150.00
-200.00
-20.00
.00
20.00
40.00
60.00
80.00
100.00
Rye Bread: (FFQ + Recall)/2

(h)

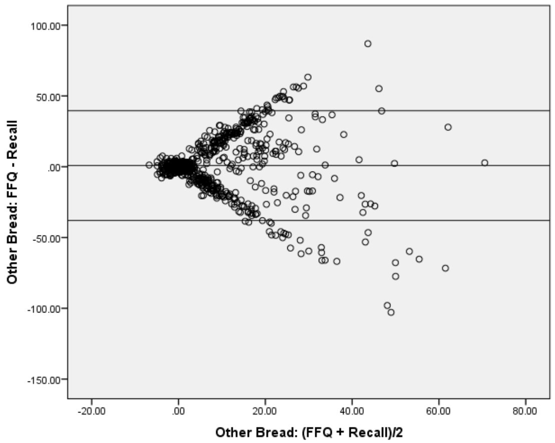

Other Bread: FFQ - Recall
100.00
50.00
.00
-50.00
-100.00
-150.00
-20.00
.00
20.00
40.00
60.00
80.00
Other Bread: (FFQ + Recall)/2

(i)

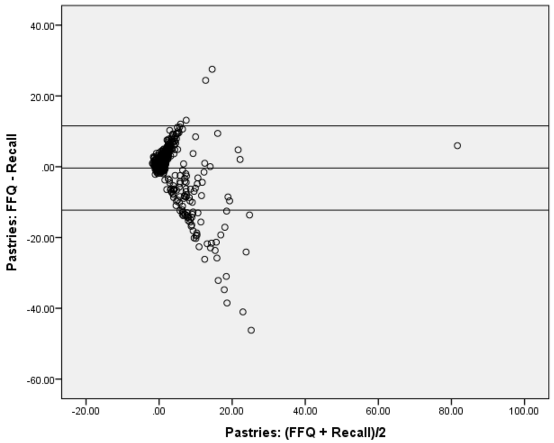

Pastries: FFQ - Recall
40.00
20.00
.00
-20.00
-40.00
-60.00
-20.00
.00
20.00
40.00
60.00
80.00
100.00
Pastries: (FFQ + Recall)/2

(j)

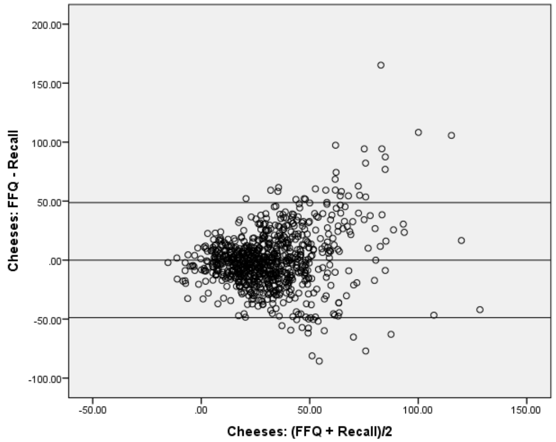

Cheeses: FFQ - Recall
200.00
150.00
100.00
50.00
.00
-50.00
-100.00
-50.00
.00
50.00
100.00
150.00
Cheeses: (FFQ + Recall)/2

(k)

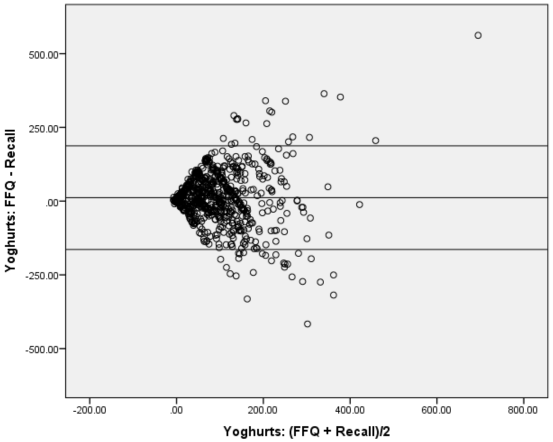

Yoghurts: FFQ - Recall
500.00
250.00
.00
-250.00
-500.00
-200.00
.00
200.00
400.00
600.00
800.00
Yoghurts: (FFQ + Recall)/2

(l)

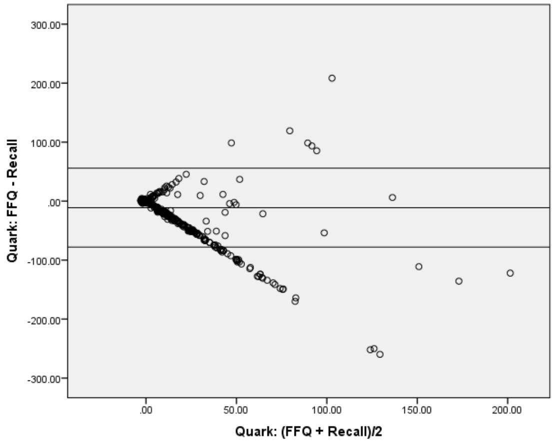

Quark: FFQ - Recall
300.00
200.00
100.00
.00
-100.00
-200.00
-300.00
.00
50.00
100.00
150.00
200.00
Quark: (FFQ + Recall)/2

(m)

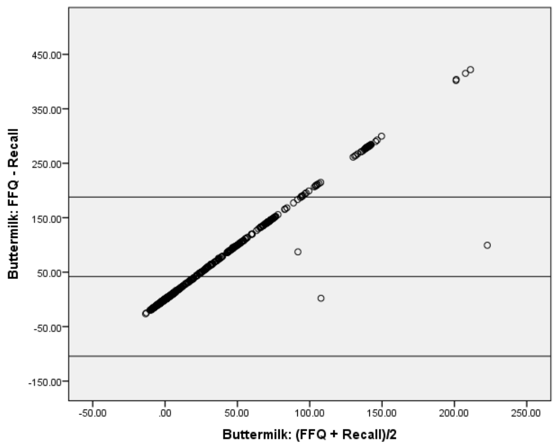
450.00
350.00
250.00
150.00
50.00
-50.00
-150.00
Buttermilk: FFQ - Recall
-50.00
.00
50.00
100.00
150.00
200.00
250.00
Buttermilk: (FFQ + Recall)/2
