# Supplementary material for: Prevalence of fermented foods in the Dutch adult diet and validation of a food frequency questionnaire for estimating their intake in the NQplus cohort
Source: BMC Nutr. 2020 Dec 3;6:69. doi: 10.1186/s40795-020-00394-z (PMC7712622; doi:10.1186/s40795-020-00394-z)

Figure S2. Bland-Altman plots demonstrating relative validity of the FFQ versus 24-h recalls for non-fermented foods. Group-level relative validity show n for: **(a)** non-fermented dairy, **(b)** butter, **(c)** cream, **(d)** ice cream, **(e)** milk, **(f)** non-fermented soya. The middle line indicates the mean difference, w hile the upper and low er lines indicate the 95% confidence intervals, respectively [calculated as: mean ± (standard deviation of the mean difference X 1.96)].

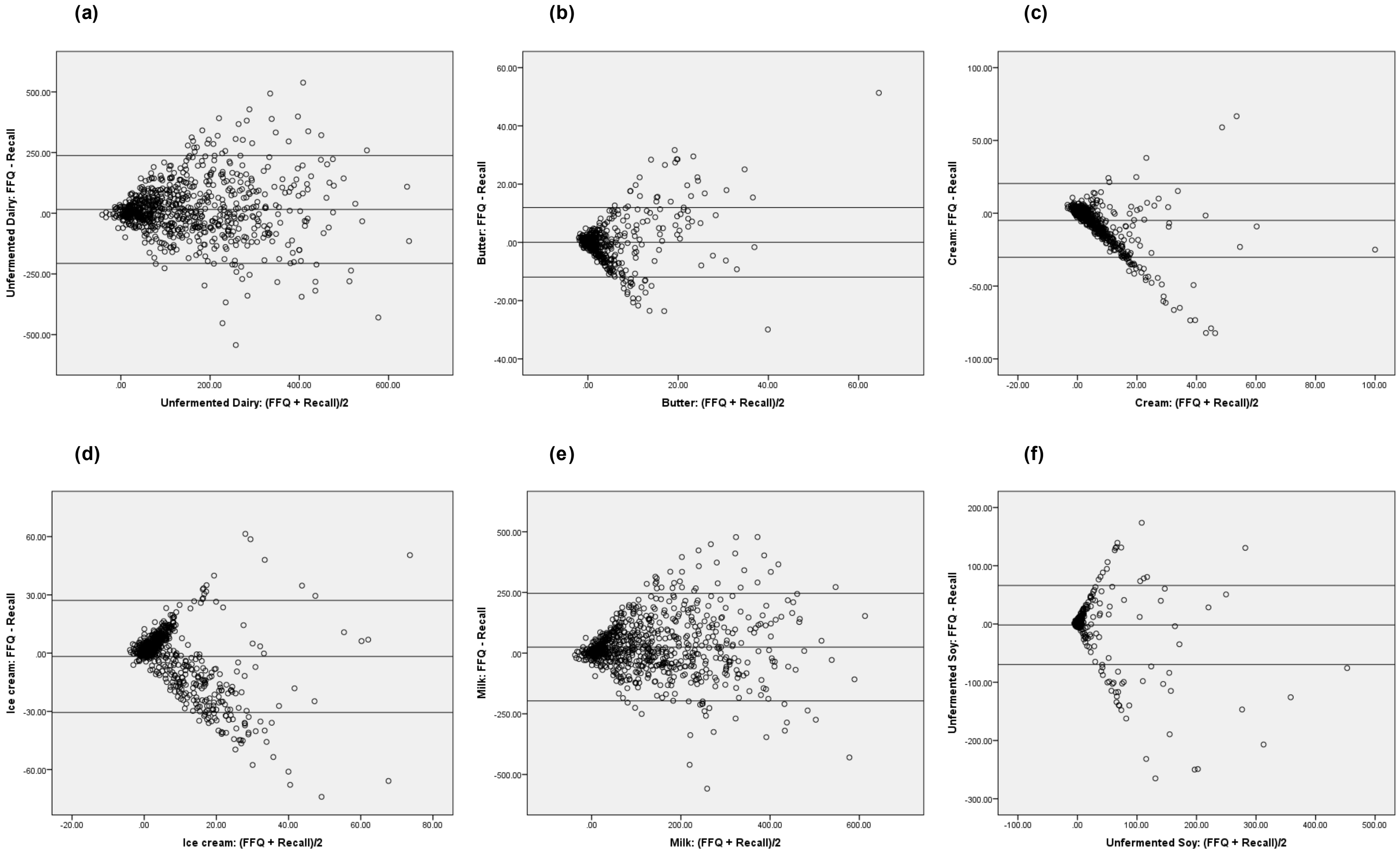

Supplement: Supplementary file 5 — Additional file 5: Figure S2. Bland-Altman plots demonstrating relative validity of the FFQ versus 24-h recalls for non-fermented foods. [file 40795_2020_394_MOESM5_ESM.pdf]
