# Supplementary material for: Prevalence of fermented foods in the Dutch adult diet and validation of a food frequency questionnaire for estimating their intake in the NQplus cohort
Source: BMC Nutr. 2020 Dec 3;6:69. doi: 10.1186/s40795-020-00394-z (PMC7712622; doi:10.1186/s40795-020-00394-z)
Supplement: Supplementary file 6 — Additional file 6: Table S4. Sex-Specific Spearman’s Correlations for Fermented and Non-Fermented Foods. [file 40795_2020_394_MOESM6_ESM.pdf]

Table S4 Sex-Specific Spearman's Correlations for Fermented and Non-Fermented Foods

	Spearman's Rank Correlation Coefficient (r_s)			
	Male (n = 425)		Female (n = 384)	
**Food Group**	**Crude**	**Energy-Adjusted**	**Crude**	**Energy-Adjusted**
Fermented beverages	0.72**	0.73**	0.80**	0.80**
Coffee	0.69**	0.69**	0.79**	0.79**
Beer	0.67**	0.66**	0.49**	0.57**
Wine	0.75**	0.69**	0.74**	0.73**
Fermented cereals/grains	0.65**	0.64**	0.64**	0.63**
Brown bread	0.27**	0.26**	0.22**	0.23**
White bread	0.26**	0.28**	0.43**	0.42**
Wholegrain bread	0.56**	0.53**	0.66**	0.64**
Rye bread	0.47**	-0.16**	0.38**	0.63**
Other bread	0.09	0.27**	0.15**	0.22**
Pastries	0.24**	0.40**	0.17**	0.24**
Chocolate	0.38**	0.38**	0.32**	0.30**
Fermented dairy	0.70**	0.70**	0.65**	0.65**
Cheeses	0.45**	0.45**	0.46**	0.43**
Yoghurts	0.58**	0.59**	0.52**	0.52**
Quark	0.14**	-0.48**	0.14**	0.39**
Buttermilk	0.12*	0.37**	0.07	-0.23**
Non-fermented dairy	0.66**	0.65**	0.70**	0.69**
Butter	0.45**	0.60**	0.52**	0.26**
Cream	0.21**	0.25**	0.18**	0.17**
Ice cream	0.22**	0.21**	0.26**	0.28**
Milk	0.64**	0.64**	0.70**	0.70**
Non-fermented soya	0.30**	-0.50**	0.46**	0.68**

FFQ, food frequency questionnaire. *, p<0.05; **, p<0.01.
